# Low-Field Benchtop NMR Spectroscopy for Quantification of Aldehydic Lipid Oxidation Products in Culinary Oils during Shallow Frying Episodes

**DOI:** 10.3390/foods12061254

**Published:** 2023-03-15

**Authors:** Miles Gibson, Benita Claire Percival, Mark Edgar, Martin Grootveld

**Affiliations:** Leicester School of Pharmacy, De Montfort University, The Gateway, Leicester LE1 9BH, UK

**Keywords:** benchtop NMR spectrometer, frying practices, cooking oils, PUFAs, MUFAs, lipid hydroperoxides, aldehyde toxins, quality control

## Abstract

Introduction: Toxic aldehydic lipid oxidation products (LOPs) arise from the thermo-oxidative deterioration of unsaturated fatty acids present in heated culinary oils when exposed to high-temperature frying episodes, and currently these effects represent a major public health concern. Objectives: In this study, we investigated the applications of low-field (LF), benchtop NMR analysis to detect and quantify toxic aldehyde species in culinary oils following their exposure to laboratory-simulated shallow frying episodes (LSSFEs) at 180 °C. Four culinary oils of variable fatty acid (FA) composition were investigated to determine the analytical capabilities of the LF NMR instrument. Oil samples were also analysed using a medium-field (400 MHz) NMR facility for comparative purposes. Results: Aldehydes were quantified as total saturated and total α,β-unsaturated classes. The time-dependent production of α,β-unsaturated aldehydes decreased in the order chia > rapeseed ≈ soybean > olive oils, as might be expected from their polyunsaturated and monounsaturated FA (PUFA and MUFA, respectively) contents. A similar but inequivalent trend was found for saturated aldehyde concentrations. These data strongly correlated with medium-field ^1^H NMR data obtained, although LF-determined levels were significantly lower in view of its inability to detect or quantify the more minor oxygenated aldehydic LOPs present. Lower limit of detection (LLOD) values for this spectrometer were 0.19 and 0.18 mmol/mol FA for *n*-hexanal and *trans*-2-octenal, respectively. Aldehydic lipid hydroperoxide precursors of aldehydic LOPs were also detectable in LF spectra. Conclusions: We therefore conclude that there is scope for application of these smaller, near-portable NMR facilities for commercial or ‘on-site’ quality control determination of toxic aldehydic LOPs in thermally stressed frying oils.

## 1. Introduction

High-field, high-resolution ^1^H nuclear magnetic resonance (NMR) analysis is extensively applied in lipidomics, together with determinations of the full composition and molecular nature of many culinary oil products (including the saturation and unsaturation status of major fatty acids (FAs) present, for example), their longevities, and even geographic origins [1]. More recently, it has also been quite widely employed for the specific determination of toxic lipid oxidation products (LOPs) in these products, including a series of aldehydes which are formed in these commonly employed cooking oils during high-temperature frying practices [2,3,4]. Furthermore, additional studies have extensively documented the potential toxicological hazards that these aldehydic species may present, along with their overall public health implications [5,6,7]. Indeed, these aldehydes, especially the α,β-unsaturated classes, are highly chemically reactive, and they form relatively stable, latent source adducts with many critical biomolecules in vivo, for example selected proteins and DNA. Such DNA damage renders these toxins mutagenic, genotoxic and, at least in some cases, carcinogenic. Consistently, Weng et al. reported that aldehydes represent the dominant carcinogens present in tobacco cigarette smoke [8]. The causal links between reactive aldehyde species and non-communicable chronic disease (NCD) risks in humans are therefore of much pertinent importance, and to date there is much evidence available indicating associations between the consumption of fried foods and the development and progression of a range of serious NCDs, for example, coronary heart diseases [9] and prostate cancer [10]. Further studies, e.g., that reported in [11], have established associations between human exposure to Chinese-style cooking fumes, which contain high levels of toxic aldehydes such as acrolein, and the risk of developing lung cancer. Moreover, it has been demonstrated that aldehydic toxins are readily transferred to foods from the oils in which they are fried; these foods typically include potato chips, beef patties, and fried chicken, which are frequently consumed by humans [12]. However, these levels were found to be significantly lower in fried potato chips than they were in the frying oil itself (only ca. 5% or so in mol/kg units), and this is attributable to only a small amount of the food mass being accounted for by uptake of aldehyde-rich oil (typically 10–15% (*w/w*) [13]), and their known chemical reactions with different classes of food biomolecules. A range of other factors such as frying oil FA compositions and frying duration are also relevant.

To date, high-field (HF) NMR spectroscopy has been successfully utilised for the rapid multicomponent analysis of quite a wide variety of LOPs present in thermally stressed frying oils, which have included primary conjugated hydroperoxydiene isomers, their secondary fragmentation products (particularly saturated and unsaturated aldehydes), and epoxy-fatty acids, for example [4,14]. Moreover, the use of two-dimensional correlation spectroscopies, both homo- and heteronuclear, has been invaluable for confirming provisional LOP assignments made in 1D spectra [15]. Notably, our research group was the very first to report these applications and advantages as early as 1994 [16]. Such advances have recently led to the development of methods for the analysis of such LOPs in fried foods, approaches which feature a key lipid extraction stage [12].

Notwithstanding, the use of both medium-field (MF, with 300–400 MHz operating frequencies) and HF ^1^H NMR spectroscopy for the simultaneous multicomponent analysis of aldehydic and further LOPs in such matrices is reliant upon a number of operational necessities such as the institutional accessibility of such expensive instruments, which are dependent on bulky cryogenically cooled superconducting magnets, the frequent use of high volumes of deuterated solvents such as deuterochloroform (CDCl_3_) for sample preparation purposes, and requirements for the professional inputs of highly specialised operational technical staff, along with the availability of those with specialist spectral interpretational skills. Moreover, the high costs of such large HF facilities (e.g., with 500–700 MHz operating frequencies), together with their stringent demands for high volumes of cryogenic cooling gases [16], limits their applications in many academic institutions, or within the commercial sector. Comparatively, low-field (LF) ‘benchtop’ NMR instruments benefit from considerably lower power requirements, permanent magnets, and virtual portability. Thus, LF NMR analysis shows great promise for applications within industrial cooking oil production, or even restaurant settings, for the direct ‘on-site’ detection and determination of edible oil quality, together with screens for toxic LOPs generated within during the use of such frying media.

Currently, such LF NMR spectrometers are emerging, cost-effective and portable alternatives to their HF counterparts [17]. Indeed, recent studies have demonstrated the growing applications of LF NMR spectroscopy to numerous analytical sectors, including ‘point-of-care’ medical diagnostics [17], forensic science, synthetic chemical reaction monitoring [18], and versatile approaches towards chemical education [19]. Furthermore, selected hyperpolarisation techniques have been applied to specific investigations [20]. The research landscape of developing applications for LF NMR has also been shown to offer major advantages in the detection of vegetable oil adulteration [21,22], together with both one- (1D) and two-dimensional (2D) NMR approaches for probing and determining edible oil authenticities [23].

Previously, preliminary studies of the LF NMR detection and analysis of aldehyde species in used and reused cooking oils has only been documented by Grootveld et al. (2014) [24,25], wherein detectable levels of selected aldehyde classes were observed in heated sunflower oil samples, and olive oil collected from a ‘real-life’ restaurant site. In the current study, we report the further exploration and development of LF NMR techniques for evaluating frying oil qualities. In particular, we consider the analytical reliability and reproducibility of LF ^1^H NMR determinations of aldehydic LOPs in oils heated according to shallow frying practices, and also limits for their detection and quantification. Comparisons of these analytical data to those acquired at MF strength are also made. We also consider the potential health risk status of these products through the reliable quantification of aldehydic LOP toxins. Overall, this LF NMR analysis technology highlights great promise for application at industrial food product manufacturing sites or restaurants for the detection and determination of these agents.

## 2. Materials and Methods

### 2.1. Reagents and ^1^H NMR Solvents

All materials, including deuterochloroform (CDCl_3_) and aldehyde calibration standards, were purchased from Sigma-Aldrich Chemical Co. (UK), unless otherwise stated; the CDCl_3_ product contained the ^1^H NMR reference standard tetramethylsilane (TMS) at an added level of 1.00% (*v*/*v*) (equivalent to 73.45 mmol/L on consideration of its density of 0.648 g/mL). NMR tubes were purchased from Norell (Morganton, NC, USA), and Eppendorf^TM^ microcentrifuge tubes were obtained from Fisher Scientific Ltd. (Loughborough, UK).

### 2.2. Culinary Oil Products and Their FA Compositions

Four culinary oils of differing triacylglycerol FA compositions were studied. These comprised a mixed-origin refined olive oil (MOO), soybean oil (SBO), rapeseed oil (RSO) and chia seed oil (CSO). With the exception of the SBO, which originated from a US retail source, all these products were purchased from reputable retail outlets based in the UK. The acylglycerol contents of FA classes present in the oils analysed were calculated using major acylglycerol resonance intensities from 400 MHz ^1^H NMR spectra of the unheated control (0 min heating time-point) samples, according to the methodological equations reported in Ref. [4].

Samples were stored under dark conditions at ambient temperature to diminish photodegradative peroxidation during storage periods prior to analysis. The lipid content profiles of each oil analysed, which are represented as % (*w/w*) saturated (SAT), monounsaturated (MUFA) and polyunsaturated (PUFA) FA contents, were found to be MOO: 16% SAT, 76% MUFA and 8% (*w/w*) PUFA; SBO: 16% SAT, 24% MUFA and 60% (*w/w*) PUFA, the latter including 5% omega-3 FAs (predominantly linolenoylglycerols); RSO: 5% SAT, 67% MUFA and 28% (*w/w*) PUFA, the latter including 7% (*w/w*) omega-3 FAs (predominantly linolenoylglycerols); CSO: 9% SAT, 9% MUFA and 82% (*w/w*) PUFA, the latter including 65% (*w/w*) omega-3 FAs (again predominantly as linolenoylglycerols). For the SBO, RSO and CSO products, omega-3 FAs were analysed via electronic integration of their characteristic linolenoylglycerol-distinctive δ = 0.95 ppm triplet resonance in 400 MHz spectra acquired on this oil.

Preliminary experiments conducted also featured a commercially available sample of a UK refined sunflower oil (SFO) product. This oil was found to have an FA content of 10% SAT, 31% MUFA and 59% PUFA.

### 2.3. Aldehydic LOP Calibration Standards

Aldehyde calibration standards were prepared using *n*-hexanal and *trans-*2-octenal in deuterochloroform (CDCl_3_) solution, and increasing concentrations of these analyte solutions were added to unheated (control) olive oil (MOO product) samples. These preparations were conducted by adding a 0.20 mL aliquot of each aldehyde calibration solution (ranging from 0.00 to 54.98 mmol/L for *n*-hexanal, and 0.00 to 40.40 mmol/L for *trans*-2-octenal), 0.20 mL of unheated olive oil, 0.20 mL of additional CDCl_3_ solvent, and 0.10 and 0.06 mL of solutions of the lipid-soluble antioxidant 2,5-di-*tert*-butylhydroquinone (2,5-DTBHQ) and the secondary ^1^H NMR chemical shift reference and internal standard 1,3,5-trichlorobenzene (TCB) (67.0 and 19.8 mmol/L, respectively), both in CDCl_3_. The densities of these aldehydes were accounted for when adding or diluting µL volumes of each. For experiments in which the signal-to-noise (STN) ratio was determined and evaluated, a corresponding series of aldehyde calibration standards were prepared in a medium without any added oil, and in these cases the 0.20 mL unheated olive oil constituent was replaced with an equivalent volume of additional CDCl_3_.

### 2.4. H NMR Analysis

^1^H NMR analysis of prepared aldehyde calibration standard solutions, and control and thermally stressed oil samples, was performed on a 60 MHz Magritek Spinsolve Benchtop system operating at a frequency of 61.67 MHz. Spectra were acquired using a 1D Proton+ sequence. Parameters employed for these analyses were 32K data points; 128 scans; acquisition time 6.4 s; repetition time 10 s; and a pulse angle of 90° (the total sample acquisition duration was ~21 min). These spectra were also acquired on a MF 400 MHz Bruker Avance AV400 NMR spectrometer (Leicester School of Pharmacy, De Montfort University, Leicester, UK) operating at a frequency of 399.93 MHz. For this facility, spectral acquisition parameters were 32K data points; 128 scans with 2 dummy scans; 3 µs pulses, spectral width 8278 Hz; and a receiver gain setting of 14.8.

### 2.5. Purity of Aldehyde Calibrations Standards Used

Reference ^1^H NMR spectra of these aldehyde standards revealed that they contained 17 and 5 mol% of their corresponding carboxylic acid oxidation products, i.e., hexanoic acid and *trans*-2-octenoic acid, for *n*-hexanal and *trans*-2-octenal, respectively, and this was again considered when preparing their calibration standard solutions; purities reported by their manufacturer were 98 and 94% (*w/w*), respectively. The identities of these carboxylic acid oxidation products were confirmed by the observation of their characteristic ^1^H NMR resonances in the aldehyde spectra acquired, i.e., that of the α-CH_2_ protons of hexanoic acid (*t*, δ = 2.354 ppm, *J* = 7.67 Hz) and those of the 2- and 3-positon olefinic protons in *trans*-2-octenoic acid (i.e., *dt*, δ = 5.831 ppm, *J* = 16.0, 1.3 Hz, and *m*, δ = 6.660 ppm, *J* = 15.9, 7.0 Hz, respectively) [26]. The concentrations and contaminating mol % values of these oxidation products in these standard solutions were determined via electronic integration of these resonances, together with the corresponding, more prominent signals arising from their parent aldehydes.

### 2.6. H NMR Determination of Different Classes of Aldehydes

Aldehyde concentrations were determined by electronic integration of their characteristic ^1^H NMR -CHO function resonances, and normalising these data to those of the total acylglycerol terminal-CH_3_ function resonance (δ = 0.82–1.11 ppm) of total FAs from the added unheated olive oil co-calibrant at both 60 and 400 MHz operating frequencies, so that aldehyde concentrations are reported as mmol aldehyde per mol of total FA (mmol/mol FA units). Aldehydic LOP levels in control and thermally stressed culinary oil products were then determined via reference to the calibration plots shown in the Results Section below. However, it should be noted that for the purpose of comparative evaluations, aldehydes were simply classified as either total saturated or total α,β-unsaturated, since it was not possible to effectively resolve superimposing resonances of individual sub-classes of these analyte species within their overall saturated and α,β-unsaturated classes at LF strength (60 MHz).

### 2.7. Calibration and Bland–Altman Dominance Plots of Authentic Standard Aldehyde Solutions in Combined CDCl_3_/Culinary Oil Media

Calibration and Bland–Altman style dominance plots of the ^1^H NMR-determined concentrations of the saturated and α,β-unsaturated aldehydes *n*-hexanal and *trans*-2-octenal, respectively, involved matched analysis sample datasets, with determinations made on these two analytes at both 60 and 400 MHz operating frequencies. Units for these determinations, which were conducted in the combined CDCl_3_/culinary oil medium, were mmol aldehyde/mol total acylglycerol FA. For these plots, Dixon’s test was applied to detect any potential outlier samples, but none were found. Similarly, all determinations which were found to have none detectable (nd, specifically values below the specified lower limit of detection (i.e., <LLOD) at both operating frequencies utilised were removed from the datasets. As recommended [27], corresponding ^1^H NMR profiles of blank samples, which were prepared as described above but with CDCl_3_ in place of culinary oils, were acquired, and their ’noise’ intensities at the appropriate δ values were included in these calibration plots. Spectra were acquired on replicate (n = 3) preparations of such blank samples for these purposes.

### 2.8. Estimation of Signal-to-Noise Ratios, and Lower Limits of Detection and Quantification Values, for Saturated and α,β-Unsaturated Aldehyde Analyte Solution Calibrants in Neat CDCl_3_ and Combined CDCl_3_/Culinary Oil Media

STN ratios for total saturated and α,β-unsaturated aldehydes were monitored by LF ^1^H NMR analysis of the *n*-hexanal and *trans*-2-octenal calibrant standards for each of these two aldehyde classes, and which were fully resolvable and quantifiable at this field strength, were determined using built-in software scripts within the *MestreNova* software package (Feliciano Barrera 9B—Bajo, 15,706 Santiago de Compostela, Spain). The signal-free region used to calculate the noise level was δ = 10.50–11.50 ppm, and Equation (1) was employed to derive the standard deviation of this value (*Noise SD*), where N = number of datapoints within the signal-free region; Y_i_ = value of each digital point in the spectrum; and Y_m_ = mean value of the digital points within that region. Mean STN ratio values were then computed as the ratio of the aldehyde resonance peak heights to that of the *Noise SD* value.

(1) $$Noise\;SD=\sqrt{\sum \frac{{\left(Y_{i}-Y_{m}\right)}^{2}}{\left(N-1\right)}}$$

Lower limits of detection and quantification (LLOD and LLOQ, respectively) were estimated as three-times and ten-times the mean STN ratio values (3(STN) and 10(STN), respectively).

### 2.9. Thermal Stressing of Culinary Oil Products According to Laboratory-Simulated Shallow Frying Episodes

Each culinary oil evaluated was exposed to laboratory-simulated shallow frying episodes (LSSFEs) for periods of 0–90 min as previously described in Ref. [12], although for the experiments outlined here, samples were collected for ^1^H NMR analysis at the 0, 30, 60 and 90 min time-points. A total of n = 3 replicate samples for each culinary oil tested and each collection time-point were obtained. Volumes (6.00 mL) of each culinary oil were placed in air-dried 250 mL glass beakers within a thermostatted silicon oil bath, which was heated to 180 °C in the presence of atmospheric O_2_ according to our LSSFEs in order to simulate shallow frying conditions. Aliquots (0.25 mL) of these oils were sampled for ^1^H NMR analysis at each of the above time-points.

### 2.10. Sample Collection and Preparation for ^1^H NMR Analysis

Aliquots (0.20 mL) of all oil samples collected were transferred to 1.5 mL microcentrifuge tubes, and then 0.40 mL of CDCl_3_ containing 1% (*v*/*v*) TMS as a chemical shift reference (δ = 0.00 ppm), and 0.06 and 0.10 mL volumes of CDCl_3_ solutions of TCB (67.0 mmol/L) and the chain-breaking antioxidant 2,5-DTBHQ (19.8 mmol/L), respectively, were added. 2,5-DTBHQ was included in these preparations in order to prevent any artefactual peroxidation of culinary oil unsaturated FAs (UFAs) during periods of sample preparation and storage prior to analysis. Subsequently, samples were vortexed thoroughly and transferred to 5 mm diameter Norell NMR tubes for ^1^H NMR analysis.

^1^H NMR analysis of these oil samples was performed at both 60 and 400 MHz operating frequencies as described in Section 2.4

### 2.11. ANOVA Model for the Statistical Analysis of Experimental Datasets

Univariate statistical chemometrics analysis was conducted to detect any significant differences between the mean replicate values of both total saturated and total α,β-unsaturated aldehydic LOPs for each oil product evaluated, each LSSFE sampling time-point (60 versus 90 min) and for each instrumental operating frequency (60 versus 400 MHz) by an analysis-of-variance (ANOVA) model. The experimental design employed comprised a three-factor model with the fixed effects of culinary oil product (O*_i_*), LSSFE sampling time-point (T*_j_*) and spectrometer operating frequency (F*_k_*). Also incorporated in the designs were first-order oil product × sampling time-point, oil product × spectrometer operating frequency, and sampling time-point × spectrometer operating frequency interaction effects (OT*_ij_*, OF*_ik_* and TF*_jk_*, respectively). The mathematical model for this design is displayed in Equation (2), where y*_ijkl_* represents each replicate aldehyde concentration, μ the overall sample mean aldehyde concentration in the absence of any possible explanatory sources of variation, and e*_ijkl_* fundamental error. An ANOVA rather than an analysis-of-covariance (ANCOVA) model was selected for this analysis since the latter approach is dependent on linear relationships between response variables (aldehyde concentrations) and the quantitative sampling time-point covariable considered, and this was clearly not the case for the dataset acquired in this study, since it is well known that all such relationships are sigmoidal (S-shaped) and not linear [12]. Software module options utilised for the performance of these ANOVA models were those available from *XLSTAT2020* (Addinsoft, Paris, France). Post hoc evaluations of the different oil products tested involved comparisons of their mean values using the Bonferroni test.

y*_ijkl_* = μ + O*_i_* + T*_j_* + F*_k_* + OT*_ij_* + OF*_ik_* + TF*_jk_* + e*_ijkl_* (2)

Subsequent to application of this model, if all first-order interaction terms were found not to be statistically significant (as indeed they were for the total unsaturated but not the saturated aldehyde outcome variables), they were removed from the model, and their variance contributions were then transferred to that of the error term for the re-testing of the main factor effects only (Equation (3)).

y*_ijkl_* = μ + O*_i_* + T*_j_* + F*_k_* + e*_ijkl_* (3)

Any missing data from the above ANOVA models were estimated by replacement with group mean or mode values, followed by reduction of error mean square degrees-of-freedom values by the number of such replacements made accordingly.

### 2.12. Computational Simulations of the ^1^H NMR Spectra of Solution-Phase Triacylglycerols

Simulated ^1^H NMR spectra of triacylglycerols, and that of their glycerol backbone, were obtained using *Bruker TopSpin NMR-SIM* software. Chemicals shift and coupling constant values, and example ^1^H NMR spectra, were first obtained from the *Human Metabolome Database* (HMBD) [26]. A 400 MHz reference spectrum of glycerol in hexadeuterated dimethylsulphoxide (d6-DMSO), was first simulated, and these data were then applied and optimised to simulate the experimental spectra of a typical triacylglycerol species at both 60 and 400 MHz operating frequencies.

## 3. Results

### 3.1. H NMR Analysis of Major Acylglycerol FA Classes in Control (Unheated) Culinary Oils at 60 and 400 MHz Operating Frequencies

**Figure 1:**
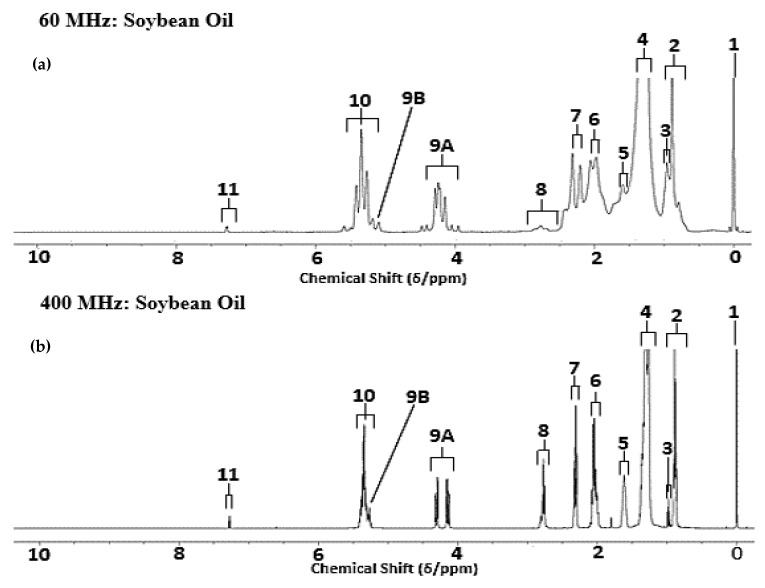

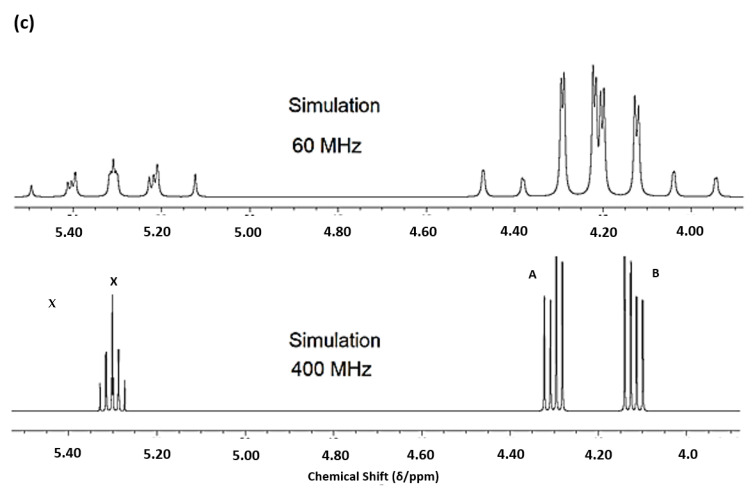
Comparative evaluation of the ^1^H NMR spectral profiles of bulk major lipids in an unheated (control) sample of SBO acquired at (**a**) 60 and (**b**) 400 MHz operating frequencies. Assignment labels for resonances are available in Table 1. (**c**) Simulated partial ^1^H NMR spectrum of the glycerol backbone resonances of a typical triacylglycerol species (expanded Resonance 9A and 9B regions (3.90–5.50 ppm)). Resonances A and B had centralised chemical shift values of 4.22 and 5.30 ppm for this simulation. The 2JAB coupling constant utilised was 10.8 Hz, with 3JAX = 6.0 Hz, and 3JBX = 5.2 Hz.

The ^1^H NMR profiles of major acylglycerols present in control (unheated) culinary oil samples were obtained for all products tested, and the 60 MHz spectra obtained were more than adequate to allow determinations of different types of FAs present, specifically SATs, MUFAs and PUFAs. Although the terminal-CH_3_ signal of ω-3 FAs (δ = 0.96 ppm) was at least partially distinguishable from that of other PUFAs (predominantly ω-6 FAs), MUFAs and SATs (δ = 0.91 ppm) at an operating frequency of 60 MHz, its electronic integration and quantification was precluded in view of resonance overlap with the latter more intense corresponding bulk lipid signal. Figure 1 shows typical spectral profiles of soybean oil (0.00–10.00 ppm), and characteristic resonances assigned and labelled are listed in Table 1, including those arising from oleoylglycerols, linoleoylglycerols and linolenoylglycerols, together with saturated FAs.

Although the 400 MHz ^1^H NMR profile shows a clear AB portion of the ABX multiplet (ABX) of its triacylglycerol glycerol backbone -CH_2_OCO- resonance pattern (δ = 4.11–4.32 ppm), at 60 MHz the appearance of this multiplet is significantly different, and this observation is attributable to the well-known ‘roofing’ effect that occurs when scalar *J*-coupled signals appear just a few Hz away from each other in spectra acquired. Whilst the chemical shift difference between the A and B signals of the glycerol backbone-CH_2_OCO- group are the same at 60 and 400 MHz when measured in ppm, when determined in Hz, the difference in chemical shift between these markedly differs between these two operating frequencies (there are only 60 Hz per ppm at 60 MHz, but 400 Hz per ppm at 400 MHz). Therefore, the signals of A and B in this ABX system are spectrally ‘closer’ in Hz when measured at 60 MHz (15 Hz apart) compared to when spectra are acquired at 400 MHz (73 Hz apart). Given that the 2*J* AB scalar coupling for the two magnetically inequivalent -CH_2_OCO- protons is 10.8 Hz, the chemical shift separation of these A and B protons in Hz is very similar to the size of the coupling constant between them at 60 MHz, and therefore this resonance appears as a distorted second-order multiplet. However, at 400 MHz, the separation between the chemical shift values of these protons is much larger than the coupling between them (i.e., a 73 Hz separation); therefore, at this MF strength, the multiplet appears as a ‘classical’ doublet of doublets for both the A and B portions of it. The low intensity signals located both slightly downfield and upfield of the multiplet at 60 MHz operating frequency (δ = 4.11–4.32 ppm) are therefore not unexpected, and have been be accurately calculated and simulated in this work, as shown in Figure 1c.

### 3.2. Detection and Quantification of Distinctive Aldehyde Classes at Different ^1^H NMR Operating Frequencies

The original experimental design for this investigation primarily featured the heating of culinary oils according to LSSFEs at a temperature of 180 °C for durations of 0, 30, 60 and 90 min. However, although reliable aldehyde levels were provided at an operating frequency of 400 MHz, the majority of aldehyde levels in all cooking oils explored at the 30 min sampling time-point were below, or far below the LLOQ value determined for the LF 60 MHz spectrometer employed, and therefore we elected to completely remove the 30 min dataset from this investigation. Hence, the remaining sampling time-points for this investigation were the 0, 60 and 90 min heating exposure durations only. However, little or no aldehydic LOPs were detectable in the oil samples at the zero-control (unheated) time-point, even at an operating frequency of 400 MHz.

**Figure 2:**
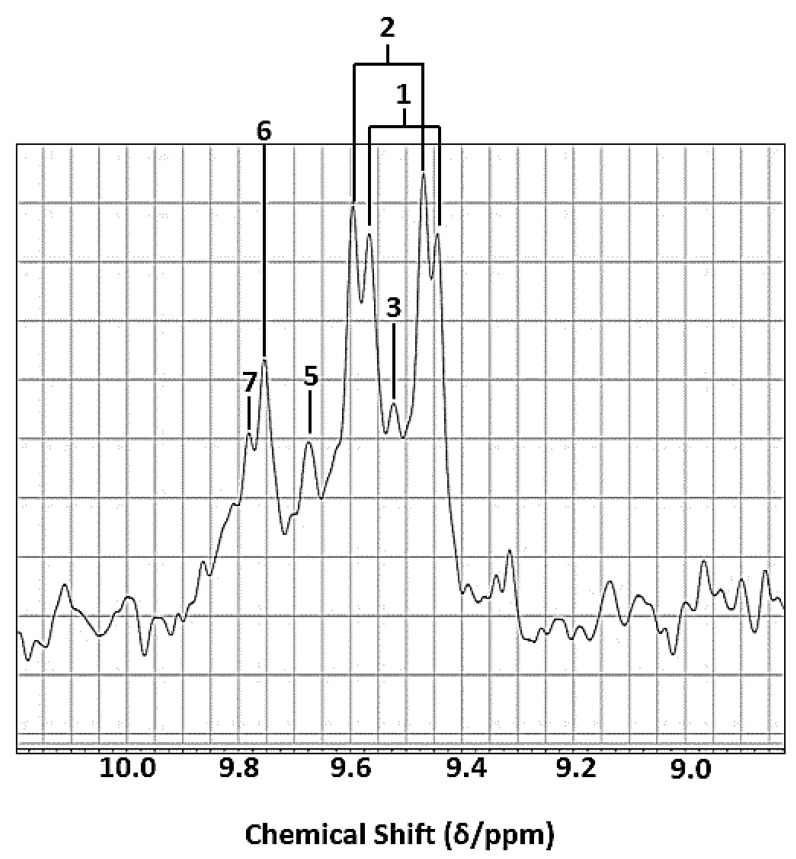
High-frequency (8.10–10.20 ppm) aldehydic proton region of the LF (60 MHz) ^1^H NMR spectrum of SBO heated at 180 °C for a 90 min duration. Numbered label assignments correspond to those in Table 2. Aldehyde signals labelled 3 and 5 represent only one line from the doublet resonance usually observed for these α,β-unsaturated aldehydes at MF strength (400 MHz), as shown in Figure 3.

Aldehydic LOP species arising from the thermo-oxidation of UFAs in edible oils exposed to laboratory-simulated shallow frying episodes were readily detectable by LF ^1^H NMR analysis, and the major signals identifiable by ^1^H NMR analysis predominantly arose from *trans*-2-alkenals, *trans-trans-*2,4-alkadienals and *n*-alkanals (Figure 2). Although this 60 MHz technique was able to partially distinguish between these two major α,β-unsaturated aldehydes generated (doublets located at δ = 9.48 and 9.52 ppm, respectively), their individual quantification using this operating frequency was not possible. However, these two unsaturated aldehyde signals were indeed resolvable from that of *n*-alkanals (δ = 9.74 ppm, *t*) at this operating frequency [12]. Additionally, signals assignable to 4,5-epoxy-*trans*-2-alkenals, *cis,trans*-alka-2,4-dienals and low-molecular-mass *n*-alkanals in thermally stressed PUFA-rich oils investigated [12] were also observable in 60 MHz spectra, most especially at the 90 min heating time-point (Table 2), but again estimates of their individual concentrations were not feasible in view of the superimposition of their resonances with themselves and/or other aldehydic LOPs.

**Figure 3:**
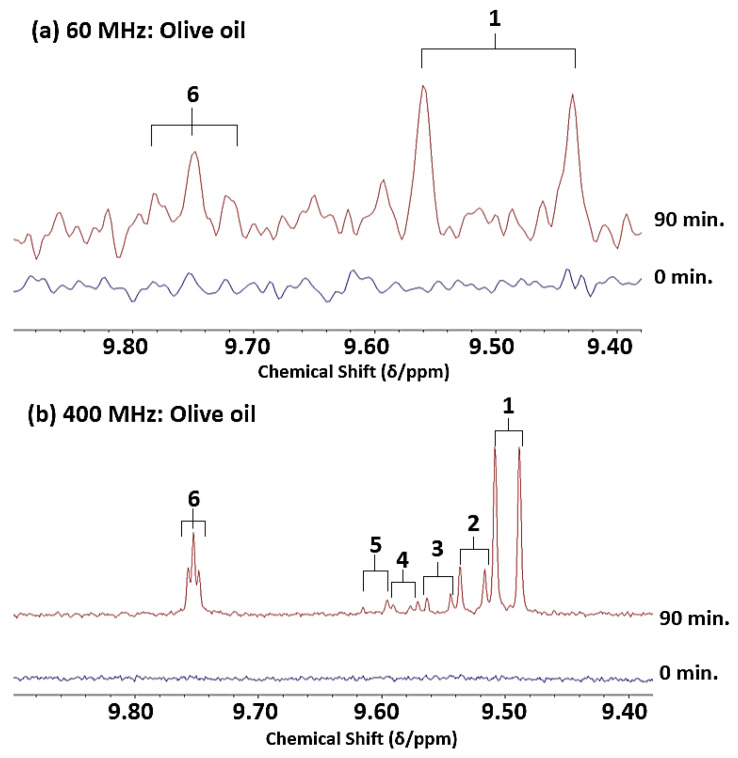
High frequency (8.10–10.20 ppm) aldehydic proton (-CHO) regions of the LF (60 MHz) and MF (400 MHz) ^1^H NMR spectra of an olive oil (MOO) sample before and after heating for a period of 90 min at 180 °C. Numbered label assignments correspond to those available in Table 2.

Notwithstanding, as expected, 400 MHz ^1^H NMR analysis had the power to not only resolve the above 9.48 and 9.52 ppm α,β-unsaturated aldehyde resonances, but also both detect and resolve the lower intensity unsaturated aldehyde resonances present, specifically those of 4,5-epoxy-2-alkenals (δ = 9.55 ppm, *d*), *cis,trans*-alka-2,4-dienals (δ = 9.63 ppm, *d*), *cis*-2-alkenals (δ = 10.07 ppm, *d*), and a composite 4-hydroxy-*trans*-2-alkenal/4-hydroperoxy-*trans*-2-alkenal signal region (δ = 9.57 and 9.58 ppm, respectively, *d)*, along with those assigned to 4-oxo-*n*-alkanals (δ = 9.78 ppm, *t*) and low-molecular-mass *n*-alkanals (δ = 9.79 ppm, *t*) [2,3,4,12,13,15,25]. Although selected resonances were visible and hence detectable, despite their decreased resolution and relatively low concentrations present in these thermally stressed oil media, these resonances were not quantitatively distinguishable from those of the more highly intense aldehydic signals in the 60 MHz spectra acquired, nor each other, and hence this series of aldehydes were not directly quantifiable at this field strength. As expected, the LF spectra acquired contained much spectrally broader overlapping resonances than those observed at the 400 MHz operating frequency. Comparative 60 and 400 MHz ^1^H NMR profiles of the MOO product prior and subsequent to thermal stressing at 180 °C for 90 min are shown in Figure 3.

The minor oxygenated aldehydic LOPs detectable and quantifiable at MF strength are only generated from PUFA sources, along with much higher and higher concentrations of the di-unsaturated aldehydes *trans,trans*- and *cis,trans*-alka-2,4-dienals, respectively. Although MUFA-rich culinary oils such as the MOO product evaluated here, which contain only low levels of PUFAs, generate markedly lower levels of aldehydes in general (a consequence of the relative resistivity of MUFAs to peroxidation), higher or much higher proportionate concentrations of *n*-alkanals and *trans*-2-alkenals than these isomeric alka-2.4-dienals are detectable therein when they are exposed to thermal stressing episodes. Therefore, for this MOO product, the intensity of the total unsaturated aldehyde spectral region is largely but not exclusively ascribable to *trans*-2-alkenals. Moreover, because the oxygenated aldehydes 4,5-epoxy-, 4-hydroxy- and 4-hydroperoxy-*trans*-2-alkenals can also arise from the thermally induced transformation of *trans,trans*-alka-2,4-dienals [4], even higher levels of these secondary LOPs are generated in PUFA-rich rather than PUFA-deplete culinary oils when exposed to such high-temperatures.

On consideration of the above aldehyde signal resolution problems encountered at 60 MHz, and for the clarity of comparative evaluations of 60 versus 400 MHz ^1^H NMR quantitative determinations, we elected to group the resonances of aldehydic LOP species into only two major classification groups, specifically total α,β-unsaturated and saturated aldehydes, and electronically integrated their total grouped signal intensities at both 60 and 400 MHz operating frequencies. Spectral regions for these two aldehydic LOP classes were specified as the α,β-unsaturated aldehyde (δ = 9.40–9.70 ppm), and the saturated aldehyde integral domains (δ = 9.70–9.80 ppm). However, the *cis*-2-alkenal doublet resonance located at δ = 10.07 ppm in MF and above spectra [12] was excluded from the grouped α,β-unsaturated aldehyde resonances in order to avoid any complications arising from its lack of spectral response in the LF spectra acquired. Additionally, although only a minor issue, these ppm ranges were also used to further negate any issues of cross-species integration observed at MF (400 MHz), for example the partial superimposition of the 4-hydroxy- and 4-hydroperoxy-*trans*-2-alkenal signals observed at this higher operating frequency.

As noted in Section 2, total α,β-unsaturated and saturated aldehyde signals were electronically integrated and then normalised to the complete δ = 0.82–1.11 ppm region, which encompassed the bulk lipid chain terminal-CH_3_ resonances of all acylglycerol FAs, including ω-3 acyl FAs. Therefore, each class of aldehyde integrals (total α,β-unsaturated and saturated) was computed to determine aldehyde concentration per mole of total FA (mmol aldehyde/mol FA).

### 3.3. Analytical Calibration of Standard Authentic Aldehydes at 60 and 400 MHz Operating Frequencies

**Figure 4:**
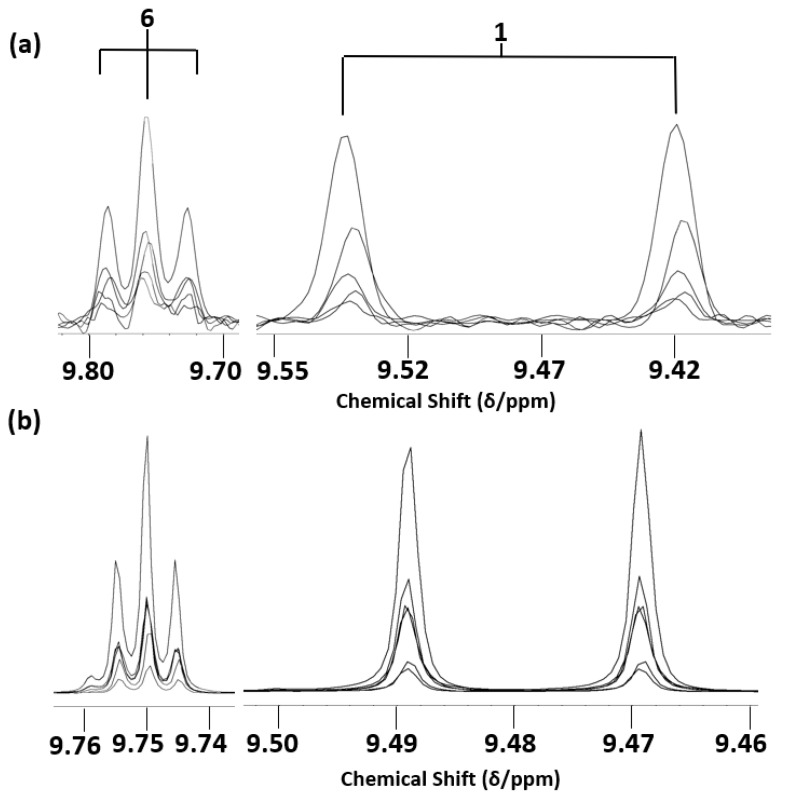
Partial aldehydic proton (-CHO) regions of the ^1^H NMR spectra of n-hexanal (δ = 9.75 ppm, *t*) and *trans*-2-octenal (δ = 9.48 ppm, *d*) in a combined CDCl_3_/unheated MOO medium at analyte solution concentrations of 5.40–54.98 and 2.42–40.40 mmol/L, respectively. Spectra acquired at (**a**) 60 and (**b**) 400 MHz operating frequencies are shown. The analyte media for these experiments were prepared according to one of the methods described in Section 2.3, and contained 0.20 mL of control (unheated) olive oil in a total volume of 0.76 mL. Numbered label assignments correspond to those available in Table 2.

Primarily, we explored the analytical calibration response of the ^1^H NMR signals of one saturated and one α,β-unsaturated aldehyde calibration analytes; these aldehydes, *n*-hexanal and *trans*-2-octenal, represent major products arising from the peroxidation of PUFAs [12]. Both LF and MF NMR spectra acquired on series of *n*-alkanal and *trans*-2-octenal calibration standards in CDCl_3_ solution containing unheated olive oil are shown in Figure 4.

**Figure 5:**
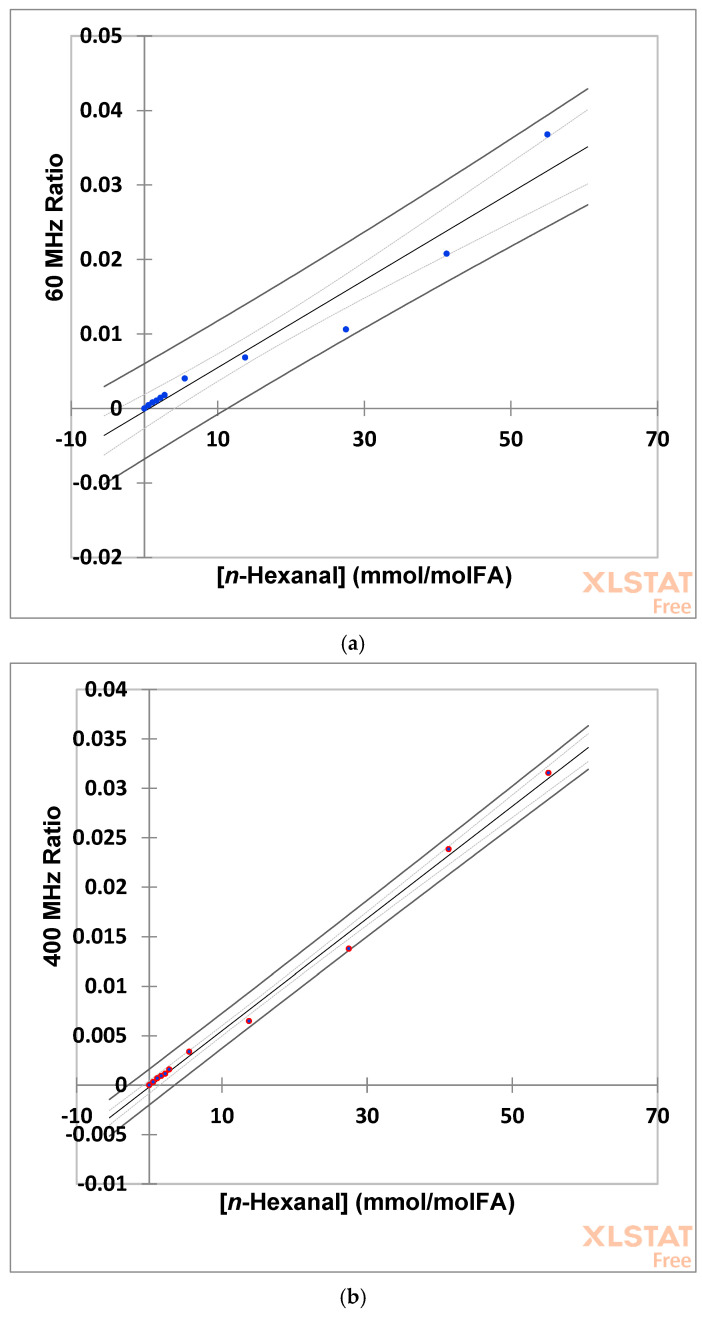

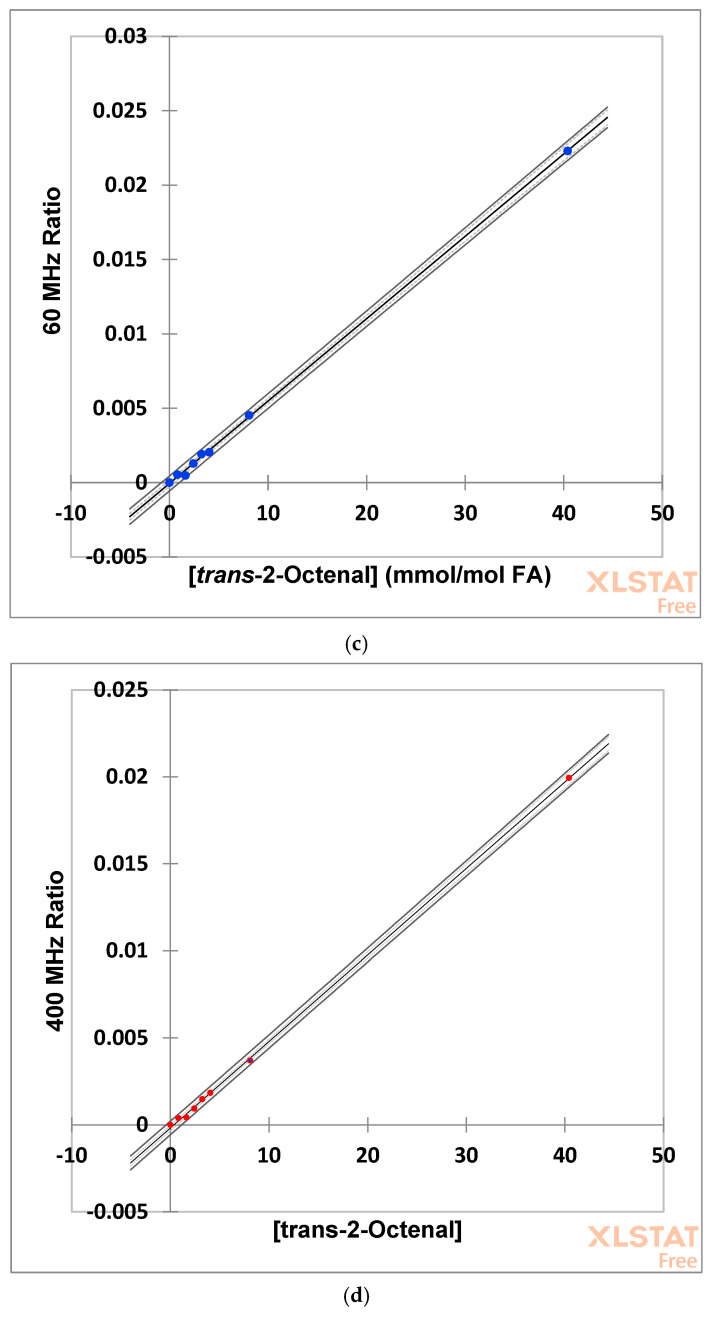
(**a**,**b**), Plots of the ratio of aldehydic proton (-CHO) intensity to that of the terminal FA-CH_3_ group of a fixed volume of an unheated (control) olive oil solution co-calibrant additive versus *n*-hexanal calibrant concentration for spectra acquired at both 60 (blue datapoints) and 400 MHz (red datapoints) operating frequencies, respectively. (**c**,**d**), As (**a**,**b**), respectively, but for the *trans*-2-octenal calibration standard. Analytical calibrant solutions contained 0.20 mL of each aldehyde stock solution (0.00–54.98 mmol/L for *n*-hexanal, and 0.00–40.40 mmol/L for *trans*-2-octenal) in CDCl_3_, 0.20 mL of the olive oil co-calibrant, a further 0.20 mL of the CDCl_3_ solvent, and 0.10 and 0.06 mL aliquots of the 2,5-DTBHQ antioxidant (67.0 mmol/L) and the additional TCB ^1^H NMR reference standard (19.8 mmol/L) solutions, also in CDCl_3_. Abbreviations for the correlation plots shown in (**a**–**d**): - - - - -, grey, 95% confidence intervals (CIs) for means; --------, black, 95% CIs for observations.

Plots of the ratio of aldehydic proton (-CHO) resonance intensity to that of the terminal FA-CH_3_ group of a fixed volume of an unheated (control) olive oil solution additive co-calibrant versus added *n*-hexanal calibrant concentration are shown in Figure 5a,b for spectra acquired at both 60 (blue datapoints) and 400 MHz (red datapoints) operating frequencies. These plots were clearly linear for this aldehyde, with Pearson correlation coefficients (r values) = 0.9757 (*p* = 3.05 × 10^−7^) and 0.9979 (*p* = 5.78 × 10^−12^) for the 60 and 400 MHz spectrometers, respectively, and 95% confidence intervals (CIs) for their regression coefficients (gradients) and ordinate (*y*-axis) intercepts were 4.88 to 6.87 × 10^−4^ and −2.65 to 1.88 × 10^−3^, respectively, for the 60 MHz, and 5.39 to 5.96 × 10^−4^ and −8.29 to 4.53 × 10^−4^, respectively, for the 400 MHz instruments (n = 10 datapoints). In view of the clear overlap of these CI values for both linear regression coefficients and y-intercept values, it may be confirmed that there were no major differences between *n*-hexanal concentrations determined by either the LF or MF NMR spectrometers employed for this analytical purpose. Moreover, the ordinate intercepts of both these calibration plots were not significantly different from zero. However, it should be noted that the 60 MHz facility’s quantitative NMR (QNMR) linear calibrant response to this analyte was not as acceptable as that achieved at MF (400 MHz).

Likewise, corresponding ratio versus calibrant concentration plots for *trans*-2-octenal yielded excellent linear relationships for both spectrometer operating frequencies (Figure 5c,d), with high r values of 0.9997 and 0.9998 (*p* = 5.34 and 2.21 × 10^−11^ for the 60 and 400 MHz instruments, respectively). The 95% CIs for their regression coefficients were 5.41 to 5.67 × 10^−4^ for the 60 MHz and 4.87 to 5.07 × 10^−4^ for the 400 MHz facilities, whereas for their ordinate intercepts, these CIs were −2.49 to 1.35 × 10^−4^ (60 MHz) and −3.28 to −0.31 × 10^−4^ (400 MHz) (n = 8 datapoints). Since the gradient parameter for the 60 MHz calibration plot was significantly greater than that observed at 400 MHz, it appears that the LF benchtop NMR instrument may overestimate this aldehydic LOP by a factor of ca. 11% when monitored as a single analyte standard in this manner, specifically calibration line gradients of 5.54 versus 4.97 × 10^−4^ for the LF and MF spectrometers, respectively. Although the 95% CIs for the 400 MHz calibration plot’s ordinate-intercept did not quite cover the zero value, the upper limit for this was very close to this expected value. Reasons for this marginally negative ordinate-intercept observed are not simply explicable However, they may arise from analyte standard preparations which are of a concentration which is slightly lower than those expected, but this is very unlikely since the same solution calibration was conducted on the 60 MHz spectrometer, and the ordinate-intercept for that plot was not significantly different from its expected zero value (Figure 5c). Despite this observation, the effect on *trans*-2-octenal’s linear calibration at 400 MHz can be considered to be largely negligible.

### 3.4. LLOD and LLOQ Values for Saturated and α,β-Unsaturated Aldehydes Monitored in Thermo-Oxidised Culinary Oils

**Figure 6:**
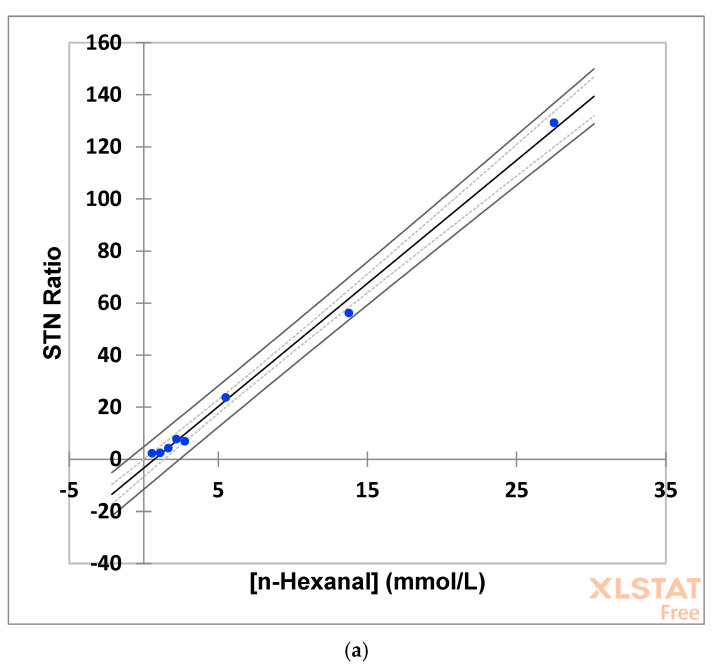

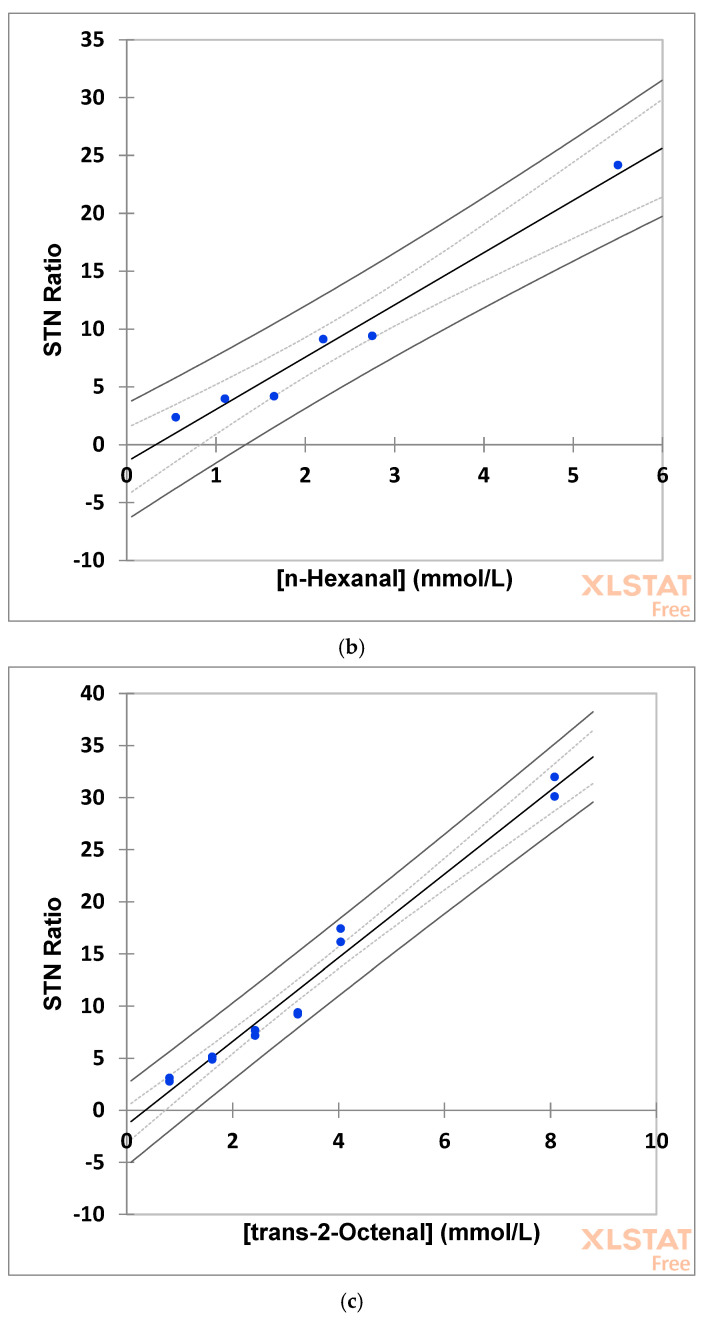

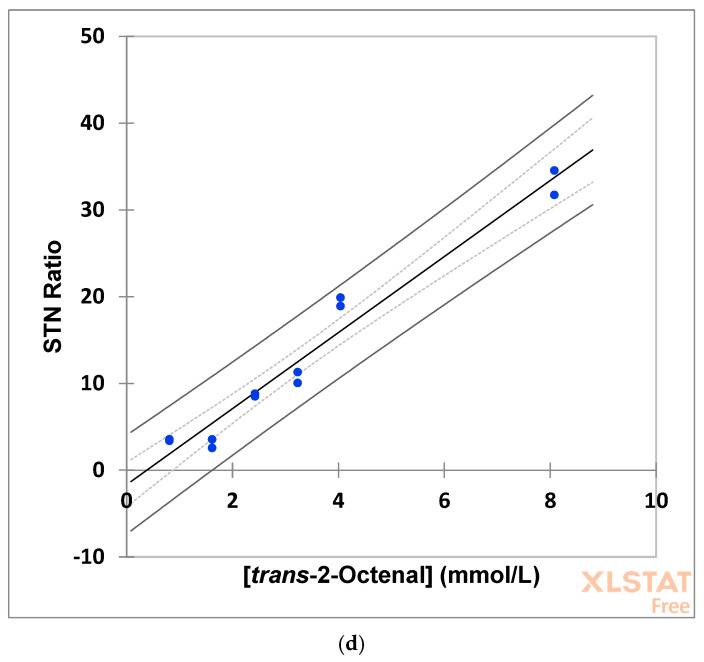
Plots of signal-to-noise (STN) ratio versus concentration of aldehyde calibrants. (**a**,**b**), *n*-Hexanal in ‘neat’ CDCl_3_ and olive oil/CDCl_3_ analytical solutions, respectively; (**c**,**d**), as (**a**,**b**), respectively, but for *trans*-2-octenal calibrant solutions. Abbreviations for the correlation plots shown in (**a**–**d**): as described in Figure 5.

Plots of STN ratio versus standard aldehyde calibrant concentration for *n*-hexanal and *trans*-2-octenal in either ‘neat’ CDCl_3_ or olive oil/CDCl_3_ analytical matrices are shown in Figure 6, and Table 3 lists simple linear regression parameters for these four plots. These results clearly show that there were no significant differences between the regression coefficients (gradients), nor ordinate- (y)-axis intercepts of these plots ‘between-aldehyde nature’ found, nor for samples which were analysed following the introduction of 0.20 mL of a culinary oil medium into the analytical matrix, as for all oil samples analysed. However, it should be noted that there were very marginally slimmer 95% CIs, together with slightly improved r values, obtained for the CDCl_3_-only analyte medium, as might be expected. Estimated LLOD and LLOQ values for these two analytes were represented by STN values of 3.00 and 10.00, respectively, and these are shown in Table 4. These estimated LLOD and LLOQ values were 0.18–0.19 and 0.62–0.65 mmol/mol FA for both classes of aldehydes analysed in CDCl_3_ media containing a 0.20 mL aliquot of olive oil.

### 3.5. Analytical Consistency of Aldehyde Calibrant Analyses at 60 and 400 MHz ^1^H NMR Operating Frequencies

**Figure 7:**
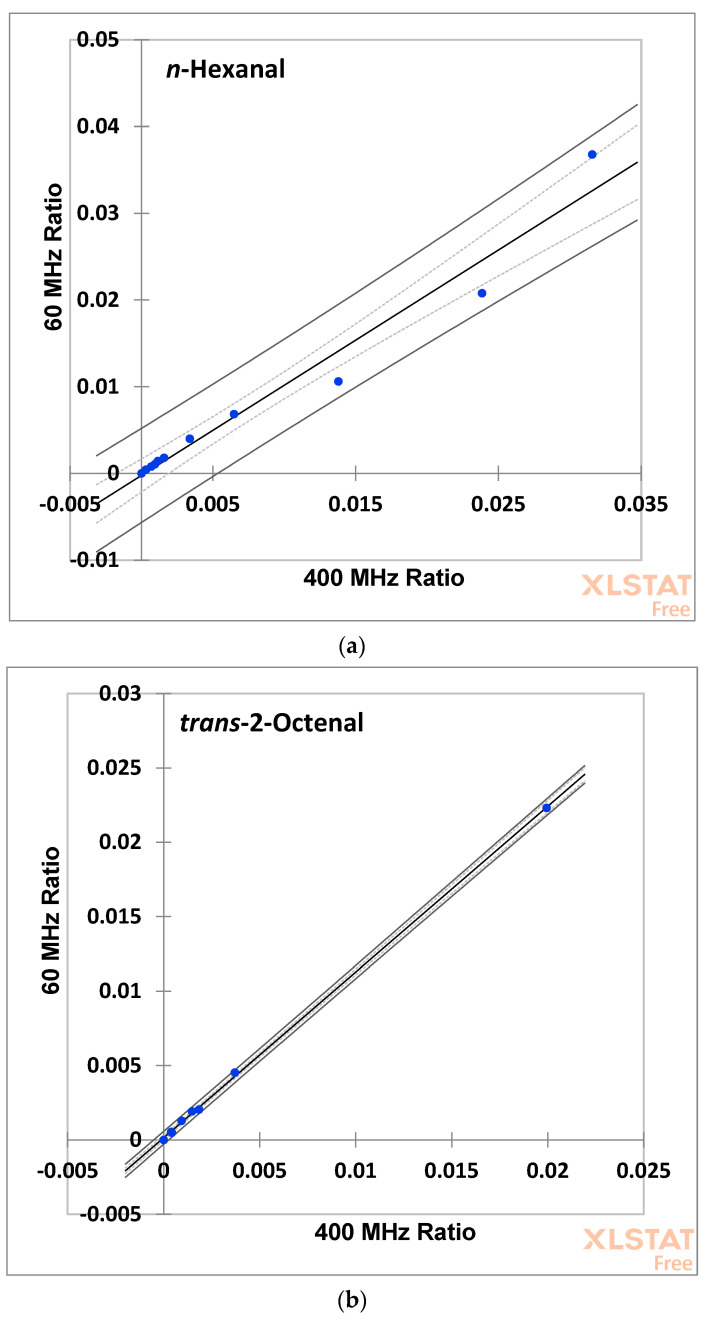

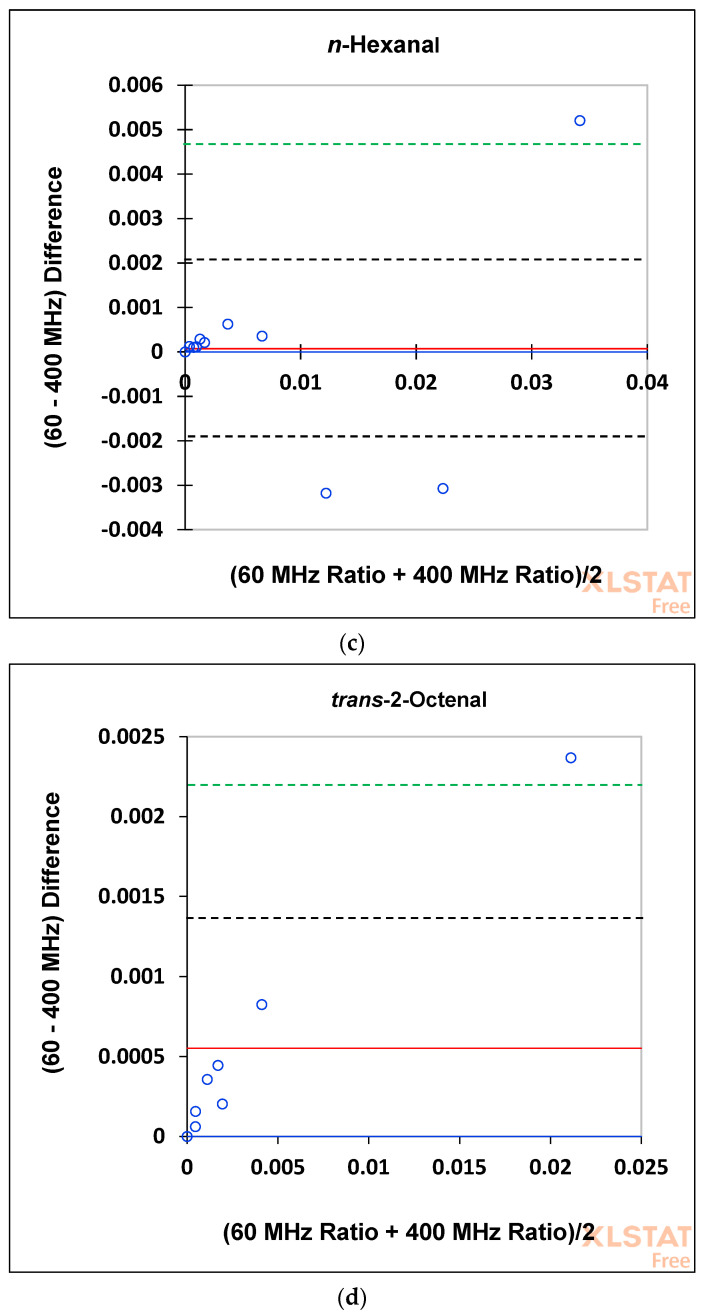
Plots of the concentrations of (**a**) *n*-hexanal (*t*, δ = 9.74 ppm) and (**b**) *trans*-2-octenal (*d*, δ = 9.48 ppm) determined at a 60 MHz operating frequency on a LF benchtop NMR spectrometer versus those obtained on a conventional MF 400 MHz NMR facility. Abbreviations for the correlation plots shown in (**a**,**b**): - - - - -, grey, 95% confidence intervals (CIs) for means; --------, black, 95% CIs for observations. (**c**,**d**), Corresponding Bland–Altman style dominance plots (BAPs) for comparisons of analytical results generated by the 60 and 400 MHz NMR facilities, respectively. For the BAP plots, the dark blue and red lines represent the null hypothesis zero difference and determined mean difference values, respectively. The dotted black lines represent 95% CIs for the mean difference (depicted as the red line), and the dotted green lines display the ±1.96s_d_ limits for the latter; s_d_ represents the standard deviation of the difference values for each plot.

The analytical consistency of aldehyde analysis conducted at the two different ^1^H NMR operating frequencies (60 versus 400 MHz) was also checked through an examination of Bland–Altman style dominance plots (BAPs) for both the *n*-hexanal and *trans*-2-octenal calibration standards (Figure 7). These BAP plots show the 400 MHz-determined concentrations subtracted from the corresponding 60 MHz ones (analytical deviations) versus the mean values of these matched sample analyses, and these provided valuable supporting information regarding the nature of operating frequency-dependent deviations between estimations of these two aldehydic analytes. For *n*-hexanal in (a), there were no apparent concentration-dependent trends for such deviations, although one datapoint laid outside the mean_d_ ± 1.96s_d_ limits, and hence overall, this analysis indicated an acceptable agreement between the two operating frequencies. For *trans*-2-octenal in plot (b), however, there appeared to be a positive deviational trend with increasing calibrant concentrations, although with only a single datapoint laying outside the ±1.96s_d_ limits. This plot also confirmed a small but nevertheless significant analytical overestimation of this aldehyde at 60 MHz, but only at the higher concentrations investigated. In view of this observation, it is proposed that a benchtop facility operating at only 60 MHz should be employed for reliable aldehyde determinations only when the aldehyde-CHO:FA terminal acylglycerol-CH_3_ signal intensity ratio values are ≤0.003 (which correspond to estimated aldehyde concentrations of ≤ca. 5.0 mmol/mol FA), so that the analytical deviations observed for measurements made at LF strength remain small.

Paired *t* tests performed on the datasets displayed in Figure 7 also verified that there was no significant ‘between-operating frequencies’ difference found for estimated concentrations of *n*-hexanal. Notwithstanding, it should be noted that *trans*-2-octenal concentrations estimated at 60 MHz were significantly greater than those determined at 400 MHz operating frequency (*p* < 10^−4^), as shown in Figure 7d; however, as noted above, this was the case only for values with relatively high abscissa axis values, which represent the mean of the integration ratios determined at both operating frequencies. Nevertheless, for this unsaturated aldehyde, the BAP showed a significant increase in the 60–400 MHz difference value with these increasing abscissa axis values. Nevertheless, at low (60 MHz Ratio + 400 MHz)/2 ratios (i.e., <0.003), these difference values were actually lower than the mean difference value determined (Figure 7d), although all were higher than the zero-control optimum. Since this deviation from the optimal zero difference optimum is approximately 0.0005 when this ratio’s values are <0.003, determinations of *trans*-2-octenal were considered acceptable within this abscissa axis limit.

### 3.6. Analytical Precision of Total Saturated and α,β-Unsaturated Aldehyde Classification Determinations in Control and Thermally Stressed Culinary Oils at 60 and 400 MHz Operating Frequencies

The analytical precision of the LF α,β-unsaturated aldehyde concentration data acquired was comparable to the results acquired at MF, with ‘between-replicate’ SD and 95% CI values for total saturated and α,β-unsaturated aldehydes being similar at these two operating frequencies. Indeed, ‘between-replicate’ coefficient of variation (CV) values for this class of aldehydes ranged from 0.4 to 13.4% (albeit with the exception of two values which were >20%) at LF, whereas those for the MF spectrometer varied from 1.2 to 12.1% (data not shown). However, for the LF data available for total saturated aldehydes, these values were less precise (CV 8.7 to 22.2%, with one value being >30%); those for the MF spectrometer employed, however, ranged from 3.0 to 12.2%, with the majority of these values being <7%.

### 3.7. LF and MF ^1^H NMR Analysis of Aldehydic LOPs in Culinary Oils Collected during Their Time-Dependent Exposure to Thermal-Stressing Episodes at 180 °C

**Figure 8:**
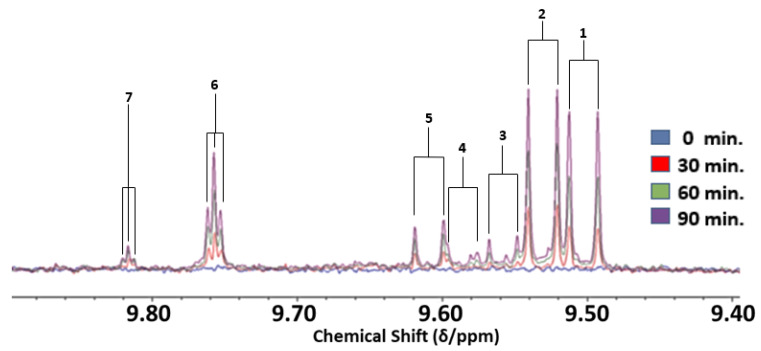
Aldehydic proton (-CHO) region (9.40–9.90 ppm) of the MF (400 MHz) ^1^H NMR spectrum of a soybean oil (SBO) sample heated for periods of 0, 30, 60 and 90 min at 180 °C according to our LSSFEs. Numbered label assignments correspond to those available in Table 2. The triplet resonance located at δ = 9.817 ppm is this spectrum is attributable to low-molecular-mass *n*-alkanal species, which arise from the thermo-oxidative deterioration of ω-3 FAs, and higher levels of these are generally characteristic of oils containing significant contents of these highly unsaturated FAs (≥5% (*w/w*)) when exposed to high-temperature thermal stressing episodes [28]. Colour key code: blue, 0 min; red, 30 min; green, 90 min; violet, 90 min.

For thermally stressed culinary oil samples collected at the 60 and 90 min LSSFE time-points, all the expected α,β-unsaturated aldehydes were readily detectable and quantifiable in thermally stressed culinary oils in spectra acquired at an operating frequency of 400 MHz (Figure 3 and Figure 8), and with the exception of the partially superimposed 4-hydroxy- and 4-hydroperoxy-trans-2-alkenal signals, all their -CHO function ^1^H NMR signals were sufficiently resolved. Importantly, at an operating frequency of only 60 MHz, the lowest determined concentration of the total α,β-unsaturated class of aldehyde at 60 MHz was found to be 0.95 mmol/mol FA (at a LSSFE time-point of 60 min), and therefore, with the exception of one missing replicate value, all 60 MHz determinations were included in the parametric ANOVA statistical analysis performed (Section 3.8).

However, unfortunately this was not the case for the total saturated aldehyde variable. Indeed, for the MOO samples, all determinations made (with the exception of a small number at the 90 min time-point), were found to be below the specified LLOQ value (Table 4), and therefore these estimates were excluded from the statistical analysis performed. Likewise, two of the SBO (both at the 60 min time-point), and four of the RSO samples (three at the 60 min, and one at the 90 min time-points), were similarly excluded. However, three further values which were below the LLOQ threshold, but were ≥90% of this parameter, were permitted to remain in the saturated aldehyde analytical dataset for data analysis by ANOVA.

Therefore, although readily observable and detectable in the 60 MHz LF spectra acquired, the majority of *n*-alkanal concentrations determined in the MUFA-rich MOO product, especially those at the earlier LSSFE sampling time-point, remained below the LLOQ threshold. Indeed, in such cases the background noise and diminished resolution and sensitivity of the LF instrument hindered the direct observation of characteristic patterns of aldehydic LOP resonances.

As expected, an enhancement of total unsaturated aldehyde formation was also observed in the PUFA-rich oils analysed on increasing the thermal stressing duration from 0 to 60 and 60 to 90 min, although the ability to distinguish between each of the aldehydic species was compromised by the low resolution at an operating frequency of 60 MHz, as noted above for the overlapping *trans*-2-alkenal and *trans-trans-*2,4-alkadienal signals (Figure 2). Comparatively, when analysed at an operating frequency of 400 MHz, *trans,trans*- and *cis,trans*-alka-2,4-dienals, 4,5-epoxy-*trans*-2-alkenals, 4-hydroxy-/4-hydroperoxy-*trans*-2-alkenals, *cis*-2-alkenals and low-molecular-mass *n*-alkanals (i.e., propanal and *n*-butanal) were all detectable and quantifiable, in addition to *trans*-2-alkenals and *n*-alkanals (Figure 3 and Figure 8). These secondary LOPs were also all observable at the 30 min heating time-point. Therefore, accurate determination of the contents of virtually all classes of both saturated and α,β-unsaturated aldehydes would require a spectrometer operating frequency of at least 400 MHz. Minor level low-molecular-mass saturated *n*-alkanal species were also observable and quantifiable at this operating frequency, However, this type of saturated aldehyde was also detectable, albeit not quantifiable, in ^1^H NMR profiles obtained on the LF spectrometer (Figure 2).

For replicate MUFA-rich MOO samples exposed to 60 or 90 min thermal-stressing periods, estimates of the total α,β-unsaturated aldehyde concentrations made at an operating frequency of 400 MHz were 2.06 ± 0.13 and 2.82 ± 0.02 mmol/mol FA, respectively (mean ± SD values), whereas at 60 MHz, these values were only 1.12 ± 0.13 and 1.70 ± 0.01 mmol/mol FA, respectively. Therefore, for this oil, total α,β-unsaturated aldehyde levels determined at LF strength were only ca. 60% of those found at MF, and this is readily explicable by the inability of the 60 MHz instrument to detect and quantify lower levels of the more structurally complex minor aldehydic LOPs generated in cooking oils exposed to LSSFEs (specifically 4,5-epoxy- and 4-hydroxy-/4-hydroperoxy-*trans*-2-alkenals, and *cis*-2-alkenals), although the major *trans*-2-alkenal class is also derived from the thermally induced peroxidation of PUFAs [12].

Although largely unquantifiable in the MOO product at a LSSFE time-point of 60 min when analysed at an operating frequency of 60 MHz, at 400 MHz mean ± SD levels of total saturated aldehydes were found to be 0.64 ± 0.06 and 0.79 ± 0.05 mmol/mol FA at the 60 and 90 min sampling time-points, respectively.

Heated PUFA-rich soybean, rapeseed and chia oils were also included in this investigation to determine the ability of LF NMR analysis to provide some level of analytical distinction between unsaturated aldehyde species generated therefrom. In view of the inability of the 60 MHz instrument to fully resolve *trans*-2-alkenal and *trans-trans*-2,4-alkadienals resonances (Figure 2), the potential application of quantitative two-dimensional (2D) ^1^H-^1^H COSY or TOCSY determinations may be valuable. Indeed, such 2D spectra are readily acquirable on LF benchtop NMR facilities, may indeed facilitate the independent quantitative determination of these aldehydes in used or fried PUFA-rich edible oils, albeit only if present at concentrations sufficiently above the specified LLOQ value (Table 4).

### 3.8. ANOVA of Total Saturated and α,β-Unsaturated Aldehyde Concentration Datasets in Culinary Oils Exposed to LSSFEs for Periods of 60 and 90 min

An extensive ANOVA model was applied to further explore the level of analytical agreement between the LF and MF spectrometers employed for the determination of both total saturated and α,β-unsaturated aldehydic LOPs, in addition to evaluations of the statistical significance of concentration differences observed ‘between oil products’ and ‘between LSSFE sampling time-points’ (i.e., at the 60 versus 90 min sampling time-points).

**Figure 9:**
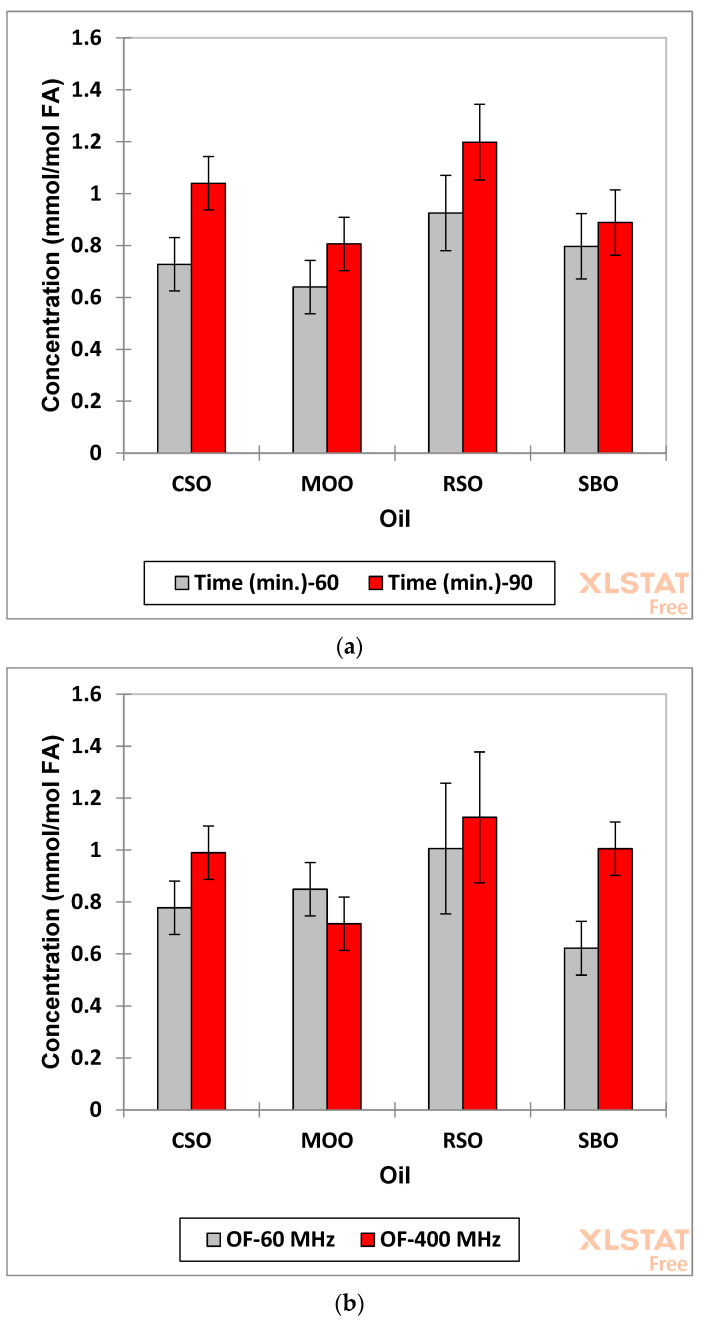

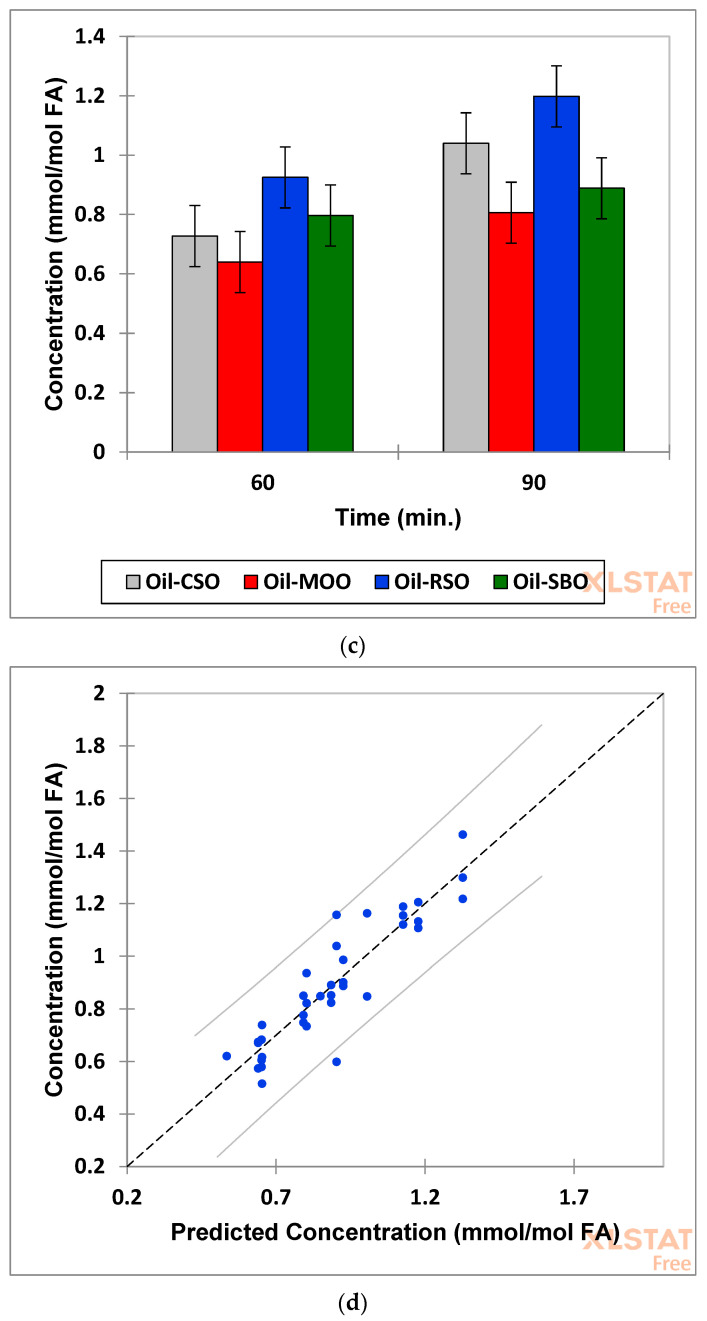
ANOVA of saturated aldehydic LOP concentration dataset from experiments involving the exposure of four different culinary oil products to LSSFEs. (**a**–**c**) Bar diagrams showing mean ± 95% CI values for saturated aldehydic LOP concentrations generated in culinary oils heated according to our LSSFEs at a temperature of 180 °C for periods of 60 and 90 min, and showing differences between heating time-points, NMR spectrometer field strength and oil products evaluated, respectively. (**d**) Plot of observed saturated aldehyde concentration versus those predicted from this ANOVA model (Equation (2)), which included a statistically significant first-order oil product x spectrometer operating frequency interaction effect (R^2^ = 0.828). Abbreviations: OF, NMR spectrometer operating frequency; CSO, chia seed oil; MOO, refined olive oil; RSO, rapeseed oil; SBO, soybean oil.

This model demonstrated that for total saturated aldehyde levels, there were highly significant differences between all the main factors investigated, with *p* = 0.036 for oil type, 0.00030 for heating time (60 versus 90 min), and 0.005 for spectrometer operating frequencies employed for the analysis; the latter observation represents higher levels observed at 400 than at 60 MHz in view of its significantly higher STN and lower LLOQ values, most notably those for certain classes of saturated aldehydes such as low-molecular-mass n-alkanals (*t*, δ = 9.78 ppm) which remain non-quantifiable at 60 MHz. Corresponding mean ± 95% confidence intervals (CIs) for this saturated aldehyde analysis protocol are shown in Figure 9. Figure 9a shows that the patterns of total saturated aldehydes were significantly lower for the 60 min heating time-point than that found at 90 min, and Figure 9c reveals that the levels of these aldehydes generated were in the product order RSO > CSO ≈ SBO > MOO.

Additionally, the oil type x spectrometer operating frequency first-order interaction effect was also statistically significant (*p* = 0.027), and this is best rationalised by again considering limits for the detection and quantification of different classes of saturated aldehyde LOPs. Indeed, Figure 9b shows that although there were higher mean values for the 400 MHz spectrometer saturated aldehyde concentrations found for CSO, RSO and SBO (those for CSO and SBO being significantly so), the extent of these differed in each case. Indeed, there was notably a quite high deviation of this parameter for the SBO product, which served as a major contribution to the statistical significance of this interaction effect. Nevertheless, this deviation is not simply explicable, although it should be noted that this oil contains a significant ω-3 FA (linolenoylglycerol) content (5% (*w/w*)), which can lead to the generation of low-molecular-mass alkanal species such as propanal and *n*-butanal following thermo-oxidation and fragmentation. It is therefore feasible that the levels of such secondary LOPs formed and remaining in the oil medium during LSSFEs are too low to be detectable and quantifiable in this oil, and hence are not included as a contribution towards its total saturated aldehyde concentration determined at both the 60 and 90 min heating time-points. This may also explain why the ‘between-operating frequency’ differences observed between the saturated aldehyde concentrations of the CSA oil product were lower than those observed for SBO, since the ω- FA content of the former was as high as 65% (*w/w*), and hence in principle it is possible that the total saturated aldehyde contents found for this oil included a significantly higher proportion of such low-molecular-mass species. However, if this was the case, a similar significant 60 MHz deviation might also be expected for the RSO product (ω-3 FA content 7% (*w/w*)), but this was not the case (Figure 9b). Moreover, an examination of both 60 and 400 MHz spectra acquired on the high ω-3 content CSA oil showed that the low-molecular-mass aldehyde resonance (δ = 9.80 ppm, *t*) had an intensity that was always <10% of that of the more intense δ = 9.75 ppm one.

A further possibility is that the LLOD and LLOQ values calculated for SBO may differ somewhat from those determined from an analyte solution containing a combined unheated olive oil/CDCl_3_ medium according to our analytical protocol described in Section 2.6 and Section 2.7; experiments to explore this are currently in progress.

The overall R^2^ value for this model with this interaction effect included was 0.828, whereas that for the model excluding all first-order interactions was significantly lower (0.716), which demonstrates the importance of including it in the model. A plot of observed total saturated aldehyde level versus that predicted from this interaction-incorporated ANOVA model is shown in Figure 9d, and this confirms an acceptable concurrence between them.

**Figure 10:**
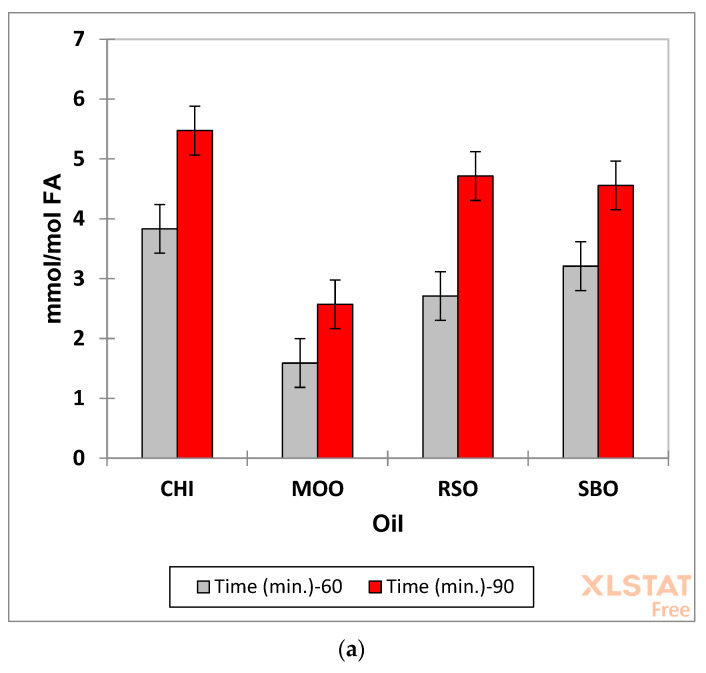

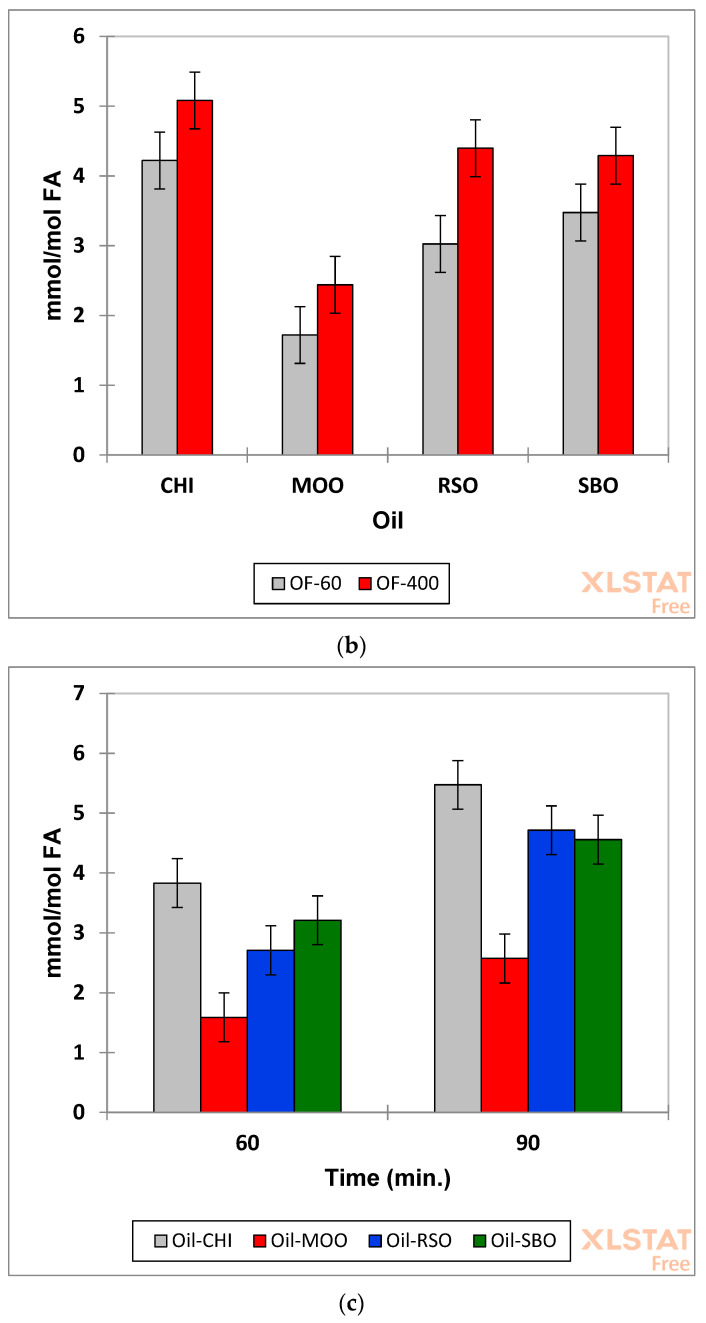

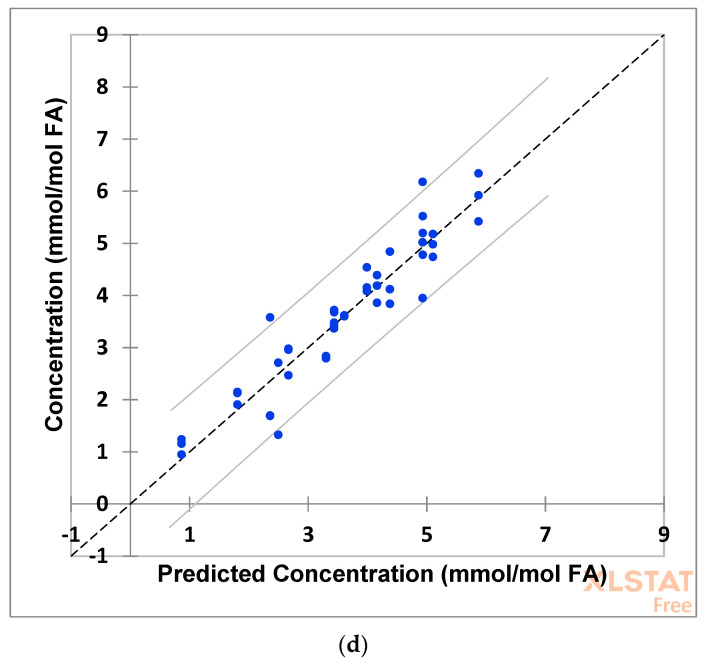
ANOVA of α,β-unsaturated aldehydic LOP concentration dataset from experiments involving the exposure of four different culinary oil products to LSSFEs. (**a**–**c**) Bar diagrams showing mean ±95% CI values for α,β-unsaturated aldehydic LOP concentrations generated in culinary oils heated according to our LSSFEs at a temperature of 180 °C for periods of 60 and 90 min, and showing differences between heating time-points, NMR spectrometer operating frequencies and oil products evaluated, respectively. (**d**) Plot of observed concentration versus those predicted from this ANOVA model (Equation (3)), which included no interaction effects (R^2^ = 0.876). Abbreviations: OF, NMR spectrometer operating frequency; CSO, chia seed oil; MOO, refined olive oil; RSO, rapeseed oil; SBO, soybean oil.

Similarly, ANOVA results for the total unsaturated aldehyde levels are displayed in Figure 10. This analysis revealed that all the main effects were again very highly statistically significant (*p* = 3.17 × 10^−14^, 1.17 × 10^−12^ and 1.49 × 10^−7^ for the ‘between-oil classes’, ‘between-heating time-points’ and ‘between-spectrometer operating frequencies’ sources of variation, respectively), whereas the mean square estimates for all second-order interactions tested were not. Therefore, all these interaction effects were removed from the model, and mean squares for the main effects were recalculated following supplementation of that for the error term with these removed, albeit insignificant contributions. The R^2^ value for the model which excluded all interaction effects was 0.876, but this value only increased to 0.907 following incorporation of these contributions. The lack of significance of these effects is clearly visible in the mean ±95% CI plots shown in Figure 10a–c, which showed similar patterns for each of the time-point, spectrometer operating frequency and oil type, respectively, with clearly higher total unsaturated aldehyde levels for the 90 min time-point, the 400 MHz operating frequency, and oil products in the order CSO > RSO ≈ SBO > MOO, as expected. The observation of higher levels of α,β-unsaturated aldehydes in the heated CSO product arose from its very high content of ω-3 FAs. Figure 10d shows a plot of observed total α,β-unsaturated aldehyde concentration versus that predicted from this interaction-free ANOVA model, and this shows a good agreement between these values.

The mean percentages of saturated and α,β-unsaturated aldehydes fromed at the 90 min LSSFE time-point for the CSO, MOO, RSO and SBO products evaluated were 16 and 84%, 23 and 77%, 20 and 80%, and 19 and 81%, respectively. Therefore, it appears that the MUFA-rich MOO product generated slightly higher proportions of saturated aldehydic LOPs over those of the other oil products when exposed to thermal stressing episodes. Moreover, the ω-3 FA-rich CSO oil appeared to produce marginally higher proportions of α,β-unsaturated aldehydes.

### 3.9. LF ^1^H NMR Detection of Aldehydic Lipid Hydroperoxide Precursors

**Figure 11:**
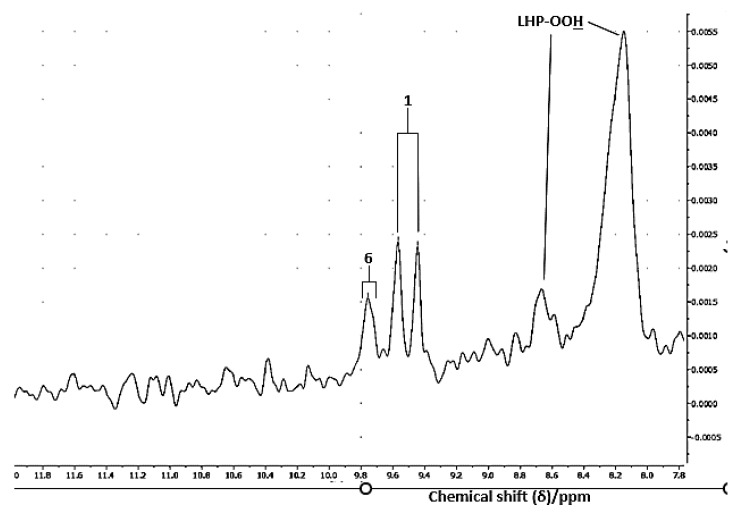
Expanded 7.80–12.00 region of the ^1^H NMR spectrum of a sample of sunflower oil thermally stressed according to our LSSFEs (180 °C for a period of 90 min). Abbreviations: LHP-OOH, broad lipid hydroperoxide signals attributable to CHPDs and/or HPMs. Numbered label assignments correspond to those in Table 2.

As previously observed in experiments conducted at both MF and HF operating frequencies, many of the LF ^1^H NMR spectra acquired here on oils exposed to LSSFEs were also found to contain broad, relatively intense signals ascribable to exchangeable lipid hydroperoxide-OOH functions, i.e., the aldehyde precursors conjugated hydroperoxydienes (CHPDs) and/or unconjugated hydroperoxymonoenes (HPMs) derived from the peroxidation of PUFAs and MUFAs, respectively (Figure 11). Indeed, these signals are reported to have solution-state chemical shift values ranging from 8.0 to 8.9 ppm in CDCl_3_ solution [2,3,4], although these values are highly dependent on analytical conditions such as analyte solution viscosity, and NMR acquisitional parameters such as NMR probe temperature for the sample, etc. Figure 11 demonstrates that 60 MHz ^1^H NMR spectra acquired on thermo-oxidised SFO samples contain one major and one more minor -OOH function resonances centred at δ = 8.08 and 8.63 ppm, respectively. As previously observed [2,3], such resonances were found to completely disappear following the addition of a small µL aliquot to the sample after thorough mixing.

Therefore, the LF ^1^H NMR monitoring of concentrations of these hydroperoxide species offers a valuable addition to the information provided by this technique on the degradation of UFAs in cooking oils, and which is a major requirement for the quality monitoring of such products [29]. Data acquired regarding changes in the ^1^H NMR-detectable levels of these primary LOPs with increasing LSSFE time-points (0–90 min) will be reported in detail in a follow-up paper.

## 4. Discussion

This study demonstrated that LF (60 MHz) ^1^H NMR analysis could detect and quantify secondary total saturated and unsaturated aldehydic LOP concentrations in thermally stressed cooking oils as a function of high-temperature LSSFE exposure time, albeit most especially at thermal stressing time-points of 60 and 90 min However, in the case of MUFA-rich oils, such as the MOO product explored here, results obtained at LF were hampered by limitations of such analysis using this operating frequency, predominantly by the lowered sensitivity of this technique and the superimposition of resonances usually resolved at MF strength, along with increased levels of spectral noise. Indeed, for the purpose of making comparative analytical evaluations between results obtained on the 60 and 400 MHz spectrometers in this study, we had no option but to combine aldehyde contents within two classification groups: total saturated and total α,β-unsaturated types. This approach was adopted despite some partial resolution between *trans*-2-alkenal and *trans,trans*-alka-2,4-dienal resonances in all oils investigated (Figure 2). However, the absolute concentrations of these two different α,β-unsaturated aldehydes could, at least in principle, be quantified on LF spectrometers through the application of suitable spectral deconvolution strategies.

As previously reported, thermally stressed MUFA-rich oils produce greater relative amounts of *trans-*2-alkenals and only minor relative levels of all other α,β-unsaturated aldehyde species [1,2,3,4,12]. However, in MF spectra acquired, resonances assignable to virtually all these unsaturated aldehydes, including isomeric alka-2,4-dienals, are observed in spectra acquired on such heated oils, and may be individually included in the resonance integration values for quantification purposes, as shown in Figure 3. However, in LF spectra, the *trans,trans-* isomer signal (δ = 9.52 ppm, *d*) is not sufficiently resolved from that of *trans*-2-alkenals (δ = 9.48 ppm, *d*), whereas levels of the *cis,trans*-isomer (δ = 9.63 ppm, *d*) generally fall below the LLOQ threshold value specified, and this served as a limitation of the technique. Therefore, these more minor aldehydic products are not accurately represented by LF ^1^H NMR data, and therefore a difference in total α,β-unsaturated aldehyde content between MF and LF analyses would be expected. This is indeed the case, with total α,β-unsaturated aldehyde concentrations in heated culinary oils ranging from 10 to 30% higher at the 400 MHz operating frequency (Figure 9b and Figure 10b). Also expected was the evolution of a more diverse range of α,β-unsaturated aldehyde compound signals in the MF spectra acquired at the extended heating periods featured (>60 min). Notably, these resonances, e.g., those of oxygenated aldehydes such as 4,5-diepoxy- and 4-hydroxy/4-hydroperoxy-trans-2-alkenals, and that of malondialdehyde, may also arise from the thermally induced oxidation and/or degradation of pre-formed alka-2,4-dienal species [12].

The higher levels of isomeric alka-2,4-dienals observed in thermally stressed PUFA-rich oils arise from the thermo-oxidative fragmentation of higher concentrations of their specific CHPD precursors, and not of HPMs from the primary MUFA peroxidation stage (the latter yielding only *trans*-2-alkeanls and *n*-alkanals on degradation). Indeed, higher total concentrations of α,β-unsaturated aldehydic species derived from PUFA-rich oils are more readily observable at LF than only *trans*-2-alkenals arising from MUFAs (Figure 10b).

Interestingly, the analytical precision of the LF α,β-unsaturated aldehyde concentration data was found to be similar to those acquired at MF operating frequency. Indeed, ‘between-replicate’ CV values determined at 60 MHz predominantly ranged from 0.4 to 13.4%, and those at 400 MHz ranged from 1.2 to 12.1%. However, for saturated aldehyde species, estimated LF CV values were found to be not as precise, whereas those at MF were predominantly <7.0%. Additionally, the CSO product displayed a less than favourable level of reproducibility for both total saturated and α,β-unsaturated aldehydes determined at the 90 min heating time-point using the LF spectrometer.

However, CSO is certainly not recommended for cooking or frying purposes anyway in view of its very high ω-3 FA content. Indeed, this oil would be expected to lead to higher levels of aldehydic LOPs than those observed in oils with high linoleoylglycerol contents (e.g., natural sunflower and corn oils); this appears to be coupled with proportionate increases in their ‘within- and between-assay’ analytical variabilities. Furthermore, the potentially higher volatilities of aldehydic LOPs arising in linolenoylglycerol-rich oils may also contribute towards this higher variance (the inclusion of this unusual oil in this study was primarily to determine the capability of LF NMR analysis to identify and determine differential classes of aldehydic LOPs arising from the specific peroxidation of omega-3 rather than omega-6 PUFAs).

The STN ratios and LLOQ values of aldehydic-CHO function proton resonances determined here are, unfortunately, restricted by the fact that only a single proton gives rise to their signals observed at 60 MHz (δ = 9.4–9.9 ppm), which are split into doublets or triplets for α,β-unsaturated and saturated *n*-alkanals, respectively. Unfortunately, the use of alternative ^1^H NMR resonances for these aldehydes is not possible, since that of the terminal-CH_3_ group of all aldehydes is obscured by very intense, major bulk lipid ones, as are those of their chain-(CH_2_)_n_- groups; the STN values of the latter are also limited by their complex coupling patterns. Similarly, the use of olefinic proton resonances of α,β-unsaturated aldehydes for QNMR purposes at LF strength is also restricted by such complex coupling patterns.

As previously described, the potential toxicological effects of dietary LOPs are likely expended by their uptake by foods fried at high-temperatures in cooking oils, followed by the consumption of such fried foods, e.g., potato chips and fried chicken, etc., by humans [7,12]. Analytical estimates of the three major classes of aldehydic LOPs in potato chips were reported to be 121 ± 33, 157 ± 43 and 126 ± 25 µmol/kg for total n-alkanals, *trans*-2- alkenals and alka-(*trans,trans*)-2,4-dienals, respectively, using ^1^H NMR analysis [12]. The mean *trans,trans*-alka-2,4-dienal level found was comparable to that determined for the single *trans,trans*-deca-2,4-dienal analyte alone by Boskou et al. [30] in French fries fried in a domestic deep-fryer at 170 °C (65 µmol/kg). It has been shown that fried food aldehyde levels are predominantly dependent on their uptake of LOP-containing frying oils during frying practices; this uptake may range from a few % to as high as 30% (*w/w*), however [7]. If we assume that this uptake level is 10% (*w/w*), a typical value for UK fast-food restaurants [13], then the above values would be ca. 10-fold higher in the oil itself, and would be equivalent to contents of 0.37, 0.49 and 0.39 mmol Aldehyde per mol Of oil FA for *n*-alkanals, *trans*-2-alkenals and *trans,trans*-alka-2,4-dienals, respectively, all of these values being lower than, but approaching, our LLOQ values for the analysis of cooking oil aldehydes by the LF benchtop NMR spectrometer employed here (Table 4). However, the total level of unsaturated aldehydes in these potato chip samples would be 0.88 mmol/mol of uptaken FA, a value which is indeed above our LLOQ threshold limit for determinations made at 60 MHz; although saturated aldehydes would not be validly quantifiable at this operating frequency, they would be detectable since the value of 0.37 mmol/mol FA remains above our LLOD limit, which is 0.19 mmol/mol FA for *n-*hexanal (Table 4). Our previously reported MF or HF ^1^H NMR analysis of aldehydic LOPs in French fry samples relies on a CDCl_3_ extraction process, and modifications to this protocol, such as the use of larger quantities of food samples for this purpose, a process giving rise to greater volumes of oils extracted therefrom, would, of course, yield lower LLOQ values for analysis at LF strength. However, it has been reported that the fried food aldehyde contents are markedly lower than those anticipated from the percentage oil uptake value alone [14], and this is likely to be attributable to their chemical consumption through Maillard or Michael addition reactions with food amino acids, peptides and proteins, and/or acetal and ketal formation on reaction with certain food carbohydrates. Indeed, it is well known that such aldehydes have a high level of reactivity with a range of biomolecules present in many biosystems. Fried food contents of aldehydic LOPs will, of course, also be strikingly dependent on a wide range of further factors, for example frying temperatures and fried food exposure times, the potential recycling of reused oils, frying type (i.e., shallow versus deep-frying episodes), frying oil MUFA, PUFA and ω-3 FA contents, and potentially also their lipid-soluble antioxidant contents, etc.

Therefore, in summary, LF NMR analysis has the capacity to monitor *n*-alkanals (saturated aldehydes) in thermally stressed culinary oils at detectable levels of 0.19 mmol/mol FA (equivalent to 0.66 mmol/L), and quantifiable at levels of 0.65 mmol/mol FA (equivalent to 2.21 mmol/L) when analysed in an olive oil co-calibrant placed in CDCl_3_ solution. Moreover, it was determined that *trans*-2-alkenal species were detectable at levels of 0.18 mmol/mol FA (equivalent to 0.63 mmol/L), with quantifiable levels of 0.62 mmol/mol FA (equivalent to 2.10 mmol/L) in an olive oil/CDCl_3_ medium. Similar LLOD and LLOQ estimates were found for both aldehyde calibrants when analysed in a ‘neat’ CDCl_3_ solution medium alone (Table 4).

Of further interest, this technological LF NMR development was also found to be valuable for the detection and quantification of precursors of the aldehydic LOPs monitored, specifically CHPDs and HPMs, as demonstrated in Figure 11. Results arising from the LF NMR assessment of the generation of these precursors, and the dependence of their culinary oil concentrations on LSSFE heating time, will be made available in a follow-up paper shortly.

This LF NMR analysis option is also valuable for determining the FA and unsaturation status of culinary oils in general, as shown in Figure 1. One interesting feature of these major lipid component H NMR profiles was differences in the spectral appearance of selected triacylglycerol multiplet resonances, and this is ascribable to the previously reported ‘roofing’ effect which occurs when scalar *J*-coupled signals appear just a few Hz away from each other in spectra acquired, as indeed they are in LF (60 MHz) spectra.

In principle, near-portable, non-stationary LF NMR spectrometers could be employed for the purpose of directly monitoring toxic aldehydic LOPs, together with their lipid hydroperoxide precursors, in a range of culinary frying oils at commercial food production and manufacturing sites, or alternatively at large outlets of global fast-food restaurant chains. Indeed, analysis is rapid, and the skillset required for the operation and management of such devices is far simpler and appealing than that required for institutionally based MF or HF spectrometers; indeed, with the exception of a succinct level of training, no previous specialist operator knowledge is required.

## 5. Limitations of the Study

Although results obtained herein have demonstrated the ability of LF benchtop NMR spectrometers to detect several different classes of aldehydic LOP species in oil samples, unfortunately quantification of these species is limited to total saturated and total α,β-unsaturated aldehydes in view of an insufficient resolution of the ^1^H NMR resonances of these LOPs, most notably that involving the latter class of these analytes. Veritably, LF NMR analysis does suffer from a number of limitations, including signal convolution which leads to issues of both identification and quantification for individual unsaturated aldehyde species. Furthermore, because of the reduced resolution and increased background noise of such instruments, the magnitude of and demands for satisfactory LLOD and LLOQ parameters were somewhat greater than those which are usually mandatory for QNMR approaches employed on MF and HF NMR facilities. Moreover, the analytical sensitivity threshold of the LF NMR technique for our analysis protocol (Table 4) also served as a further limitation; this involved the analysis of only a 0.20 mL aliquot of culinary oil in a total NMR tube volume of 0.760 mL. However, pilot experiments have revealed that we may increase this analytical volume of oil sample up to 0.40 mL, and therefore the LLOD thresholds determined here would be decreased 2-fold if that were the case. Unfortunately, larger increments in proportionate sample volume restrict signal resolution and hence the analysis of aldehydes and other LOPs in view of the resulting high viscosity of the analyte solution medium.

Data available in Figure 9b and Figure 10b revealed that for both the 60 and 90 min heating time-points, mean total saturated aldehyde concentrations determined in PUFA-rich oils at an operating frequency of 60 MHz were 79 and 89% of that determined at 400 MHz for the CSO and RSO products, respectively, but only 62% for the SBO one (these ‘between-oil’ differences in analyte receptivity were also manifested by the observation of a statistically significant first-order oil × spectrometer operating frequency interaction effect for this aldehyde classification). For total α,β-unsaturated aldehyde levels, however, corresponding values were 83, 69 and 81% for the CSO, RSO and SBO products, respectively (MOO 71%). Therefore, it is important that this current restriction should be recognised by analysts and researchers, and perhaps the application of a suitable ‘correction factor’ should be considered to overcome this issue.

It should also be noted that saturated aldehydic LOPs (mainly two types of NMR-distinguishable *n*-alkanals) are generated at much lower concentrations than the α,β-unsaturated aldehyde classifications, and for this study, it was found that the former class were only ca. 20% of the total aldehyde levels determined by the LF spectrometer at the 90 min heating time-point. These diminished concentrations will also restrict their determination at an operating frequency of only 60 MHz.

Another consequence of the LF ^1^H NMR monitoring of aldehydes in culinary oils, heated or otherwise, is that the total amounts of aldehydic LOPs liberated from the peroxidation of UFAs is not a direct reflection of those found in these oil products following exposure to thermal stressing periods. Indeed, a significant proportion of these toxins have boiling-points below standard frying temperature (now ranging from 160 to 180 °C), with some, such as acrolein (the lowest α,β-unsaturated aldehyde homologue), exceedingly so [14]. Hence, a quite substantial fraction of the total amount of aldehydic LOPs evolved from these frying oil ‘reaction’ media are in the volatile, i.e., gaseous form, and due caution should be exercised by humans to avoid the inhalation of these toxins during frying or cooking exercises, be they commercial or domestic. Therefore, oil aldehyde contents only serve as incomplete reflections of the total amounts generated.

Since our LF ^1^H NMR-determined aldehyde concentrations were segmented into total saturated and α,β-unsaturated classifications, variance contributions towards these from structurally more complex LOPs would be expected to expand with increasing complexity level, most notably for PUFA-rich oils which have been exposed to prolonged heating durations. Ultimately, saturated aldehydes were only observed at quantifiable levels in the LF NMR spectra of heated oil samples from the 60 min, but not the 30 min, time-point, although not all oils thermally stressed in this manner for a 60 min period gave sample aldehyde levels higher than the LLOQ threshold level determined here. Despite this, LF-NMR analysis shows promise as an appropriate approach for detecting and quantifying total aldehydes in oils that have been heated for durations which are ≥60 min, and putatively less so for samples with higher oil contents present in analyte media solutions.

## 6. Conclusions

In this study, we have demonstrated the applications of LF NMR analysis for the identification and quantification of both saturated and unsaturated aldehydic LOP species in heated culinary oils. This investigation was also valuable for monitoring the abilities of LF and MF NMR spectrometers to distinguish between differential classes of these toxic products.

The investigations conducted demonstrated that this technology was valuable for determining the concentrations of LOP toxins in such culinary oil products exposed to LSSFEs, but only for those which were thermally stressed for minimal durations of 60 min or so. Although distinctive patterns of aldehydes were observed for different oil products investigated, unfortunately resonance superimposition and the limited sensitivity of the technique precluded the determination of individual molecular classes of aldehyde toxins in these products. However, LF NMR analysis remains a very promising technique, and in some thermally stressed PUFA-rich oils, a total of four or more unsaturated aldehydes, and two saturated aldehydes, along with their CHPD and/or HPM precursors, were detectable, although not all were directly quantifiable (Figure 2 and Figure 11). However, the LF NMR determination of total saturated and α,β-unsaturated aldehyde oil contents, along with those of their hydroperoxide precursors, remains a relevant and acceptable avenue for aldehydic toxin analysis in commonly employed frying oils.

In principle, the non-stationary LF NMR technique could be employed for the ‘on-site’ monitoring of aldehydic toxins present in thermally stressed cooking oil samples at food manufacturing sites, or even at fast-food take-out restaurants.

## Figures and Tables

**Table 1:** Assignments for the major lipid resonances found in the ^1^H NMR spectra of the culinary oils evaluated in this study at both 60 and 400 MHz operating frequencies. The spectral ranges and multiplicities of these signals are also provided in the second column.

Resonance Assignment Code	δ/ppm (*Coupling Pattern*)	Assignment
1	0.00 (*s*)	TMS-CH_3_
2	0.85–0.92 (*t*)	Acyl chain Terminal-CH_3_ (non-ω-3 FAs)
3	0.94–0.97 (*t*)	Acyl chain Terminal-CH_3_ (ω-3 FAs)
4	1.20–1.38 (*m*)	Bulk acyl chain -(CH_2_)_n_-
5	1.56–1.66 (*m*)	Acyl chain -OCO-CH_2_-CH_2_-
6	1.95–2.11 (*m*)	MUFA/PUFA Acyl chain -CH=CH-CH_2_-
7	2.26–2.36 (*dt*)	Acyl chain -OCO-CH_2_-
8	2.75–2.82 (*dd*)	PUFA Acyl chain -CH=CH-CH_2_-CH=CH-
9A	4.11–4.32 (ABX, *dd*, *dd*)	Glycerol backbone -CH_2_-OCO-
9B	5.20–5.23 (ABX, *dd*)	Glycerol backbone -CH-OCO-
10	5.23–5.28 (*m*)	MUFA/PUFA -CH=CH-
11	7.26 (*s*)/7.27 (*s*)	TCB Aromatic Protons/CHCl_3_

**Table 2:** Assignments for the labelled aldehydic proton (-CHO) resonances present in the 60 and 400 MHz ^1^H NMR profiles of culinary oils exposed to LSSFEs for periods of 60 or 90 min in Figures 2–4 and 8 (resonance 4 was undetectable at the 60 MHz operating frequency). These assignments were made by a consideration of those available in Refs. [2,3,4,12,13,15,25]. The spectral ranges and multiplicities of these signals are also provided in the second column. Abbreviations: *d*, doublet; *t*, triplet.

Resonance Assignment Code	δ/ppm (*Coupling Pattern*)	Assignment (-CHO Signal)
1	9.47–9.51 (*d*)	(*trans*)-2-Alkenals
2	9.51–9.55 (*d*)	(*trans,trans*)-Alka-2,4-dienals
3	9.54–9.58 (*d*)	4,5-Epoxy-(*trans*)-2-alkenals
4	9.57–9.61 (*d*)	Combined 4-Hydroxy-(*trans*)-/4-Hydroperoxy-(*trans*)-2-alkenals
5	9.62–9.65 (*d)*	(*cis,trans*)-Alka-2,4-dienals
6	9.73–9.76 (*t*)	*n*-Alkanals
7	9.78–9.82 (*t*)	Low-molecular-mass *n*-Alkanals

**Table 3:** Linear regression parameters for plots of analytical STN ratio values versus added *n*-hexanal and *trans*-2-octenal calibrant concentrations in media including and excluding olive oil added to the analyte media at an operating frequency of 60 MHz. The 95% Confidence intervals (CIs) are provided for the estimated regression coefficients and ordinate intercepts of these plots. Abbreviation: r, Pearson correlation coefficient.

Aldehyde	Solution Medium	r	Regression Coefficient ± 95% CIs	Ordinate Axis Intercept ± 95% CIs
*n*-Hexanal	CDCl_3_	0.9980	4.73 ± 0.30	−3.34 ± 3.35
*n*-Hexanal	Olive Oil/CDCl_3_	0.9860	4.52 ± 1.04	−1.47 ± 2.92
*trans*-2-Octenal	CDCl_3_	0.9886	4.01 ± 0.43	−1.41 ± 1.77
*trans*-2-Octenal	Olive Oil/CDCl_3_	0.9801	4.38 ± 0.62	−1.67 ± 2.57

**Table 4:** Estimated LLOD and LLOQ values for n-hexanal and *trans*-2-octenal at an ^1^H NMR operating frequency of 60 MHz. Units of mmol/L were employed for all analyte media, but mmol/mol FA units were also applicable to those prepared in a combined olive oil/CDCl_3_ medium.

Aldehyde	Solution Medium	LLOD: 3(STN) (mmol/L)	LLOD: 3(STN) (mmol/mol FA)	LLOQ: 10(STN) (mmol/L)	LLOQ: 10(STN) (mmol/mol FA)
*n*-Hexanal	CDCl_3_	0.63	n/a	2.10	n/a
*n*-Hexanal	Olive Oil/CDCl_3_	0.66	0.19	2.21	0.65
*trans*-2-Octenal	CDCl_3_	0.75	n/a	2.49	n/a
*trans*-2-Octenal	Olive Oil/CDCl_3_	0.63	0.18	2.10	0.62

Notably, the -CHO function resonances of both of these aldehydic calibrant species did not suffer from the issue of signal collapse, and fully maintained their multiplicities at an operating frequency of 60 MHz.

## Data Availability

All study data will be provided to those who reasonably request this information from the correspondence author.

## List of Abbreviations

Analysis-of-variance (ANOVA); Analysis-of-covariance (ANCOVA); conjugated hydroperoxydiene (CHPD); chia seed oil (CSO); fatty acid (FA); unconjugated hydroperoxymonoene (HPM); laboratory-simulated shallow frying episodes (LSSFEs); lower limit of detection (LLOD); lower limit of quantification (LLOQ); lipid oxidation products (LOPs); mixed refined olive oil (MOO); monounsaturated fatty acid (MUFA); nuclear magnetic resonance (NMR); NMR spectrometer operating frequency (OF); polyunsaturated fatty acid (PUFA); quantitative NMR (QNMR); rapeseed oil (RSO); saturated fatty acid (SAT); signal-to-noise (STN); soybean oil (SBO); standard deviation (SD); unsaturated fatty acid (UFA).
